# Morphological differences between species of the sea bass genus *Lateolabrax* (Teleostei, Perciformes), with particular emphasis on growth-related changes

**DOI:** 10.3897/zookeys.859.32624

**Published:** 2019-07-02

**Authors:** Kōji Yokogawa

**Affiliations:** 1 13-5 Higashihama, Tadotsu-cho, Nakatado-gun, Kagawa 764-0016, Japan Unaffiliated Tadotsu Japan

**Keywords:** *
Lateolabrax japonicus
*, *
Lateolabrax maculatus
*, *
Lateolabrax latus
*, morphology, growth, new key

## Abstract

Morphological differences, including growth-related changes, were examined in three morphologically similar East Asian sea bass species, *Lateolabraxjaponicus*, *L.maculatus* and *L.latus*. In many cases, body measurements indicated specific patterns of growth-related proportional changes. *Lateolabraxlatus* differed from the other two species in having greater body depth, caudal peduncle depth, caudal peduncle anterior depth, snout length, and upper and lower jaw length proportions. In particular, scatter plots for caudal peduncle anterior depth relative to standard length (SL) in that species indicated complete separation from those of the other two species, being a new key character for identification. Comparisons of *L.japonicus* and *L.maculatus* revealed considerable proportional differences in many length-measured characters, including fin lengths (first and second dorsal, caudal and pelvic), snout length, post-orbital preopercular width (POPW) and post-orbital length. In particular, snout length (SNL) and POPW proportions of the former were greater and smaller for specimens >200 and ≤ 200 mm SL, respectively. Because the scatter plots of these proportions for the two species did not overlap each other in either size range, identification of the species was possible using a combination of the two characters. In addition, scatter plots of the POPW / SNL proportion (%) of *L.japonicus* and *L.maculatus* were almost completely separated throughout the entire size range examined (border level 90%), a further aid to identification. The numbers of pored lateral line scales and scales above the lateral line tended to increase and decrease with growth, respectively, in *L.japonicus*, whereas scales below the lateral line and gill raker numbers tended to increase with growth in *L.maculatus*. Because the ranges of these meristic characters may therefore vary with specimen size, they are unsuitable for use as key characters. Accordingly, a new key is proposed for the genus *Lateolabrax*.

## Introduction

**Figure 1.**
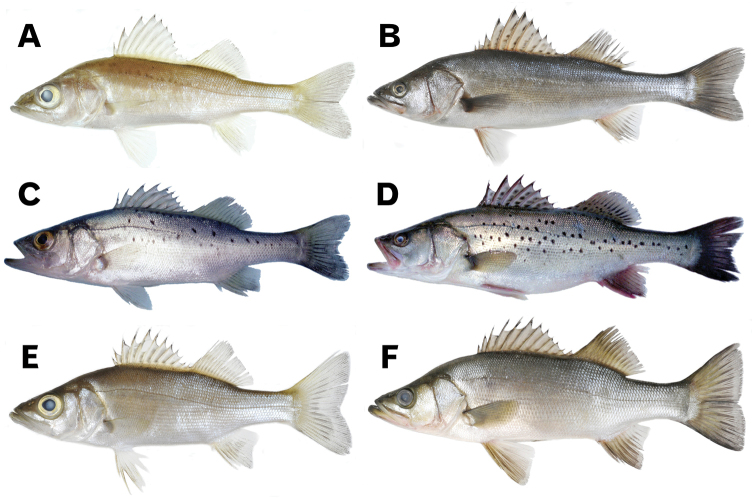
General aspects of small (fingerling) and large (adult) individuals of *Lateolabraxjaponicus* (**A, B**), *L.maculatus* (**C, D**) and *L.latus* (**E, F**) in fresh condition. **A**KPM-NI 27449 (91.9 mm SL) **B**KPM-NI 30671 (331.0 mm SL) **C** uncatalogued specimen (94.3 mm SL) **D**BSKU 100776 (265.0 mm SL) **E**KPM-NI 29044 (97.1 mm SL) **F**KPM-NI 24656 (369.0 mm SL). **A, B, E** and **F** were photographed by Hiroshi Senou (KPM), **C** and **D** were photographed by K. Yokogawa.

The sea basses of the genus *Lateolabrax* (Lateolabracidae) are common East Asian coastal marine fishes (occasionally also occurring in fresh water). Bleeker (1854–57) established the genus for a single species, *Lateolabraxjaponicus* (Cuvier, 1828), Katayama (1957) later describing a second species, *Lateolabraxlatus*, from Japan. More recently, Yokogawa and Seki (1995) concluded that differences between the Japanese and Chinese forms of “*L. japonicus*” were sufficient for the Chinese form to be recognized as a distinct species, being referred to as “spotted sea bass” by Yokogawa and Tajima (1996). Finally, it was formally redescribed as *Lateolabraxmaculatus* (McClelland, 1844) in Yokogawa’s (2013b) revision, where *Lateolabraxlyiuy* (Basilewsky, 1855), which is incorrectly treated as valid and applied to the Chinese form (Kottelat 2013; Eschmeyer 2019), was regarded as a junior synonym of *L.maculatus*. At this point, three valid species of *Lateolabrax* are recognized (Fig. 1).

*Lateolabraxlatus* has been distinguished from *L.japonicus* by having greater proportions of body and caudal peduncle depth (BD and CPD), more dorsal and anal fin rays (≥15 and ≥9, respectively), fewer scales below the lateral line (≤16) and possessing ventromandibular scale rows (VSRs) (Katayama 1957). Furthermore, the range of dorsal fin ray (DFR) counts in *L.latus*, which had been considered to not overlap that of *L.japonicus*, had become established as a key identification character (e.g., Katayama 1960a, 1965, 1984; Hatooka 1993). However, subsequent finding of *L.latus* individuals with 14 DFRs [overlapping the range in *L.japonicus* (12–14 DFRs)] (Hatooka 2000, 2013; Murase et al. 2012) made this character an incomplete key for identification. In addition, VSRs have not been adopted in recent keys proposed for *Lateolabrax* (Hatooka 2000, 2013), because they have been found in some specimens of the other two *Lateolabrax* species (Paxton and Hoese 1985; Hirota et al. 1999; Kang 2000; Murase et al. 2012). On the other hand, recent keys have included “caudal fin notch depth,” being shallower in *L.latus* than in the other two species (Hatooka 2000, 2013), although the lack of any proportional information means that verification following examination of possible growth-related changes is necessary. Furthermore, proportional differences in BD and CPD appear to be based on the premise that their proportions are stable (isometric growth), although this has not been verified to date.

**Figure 2.**
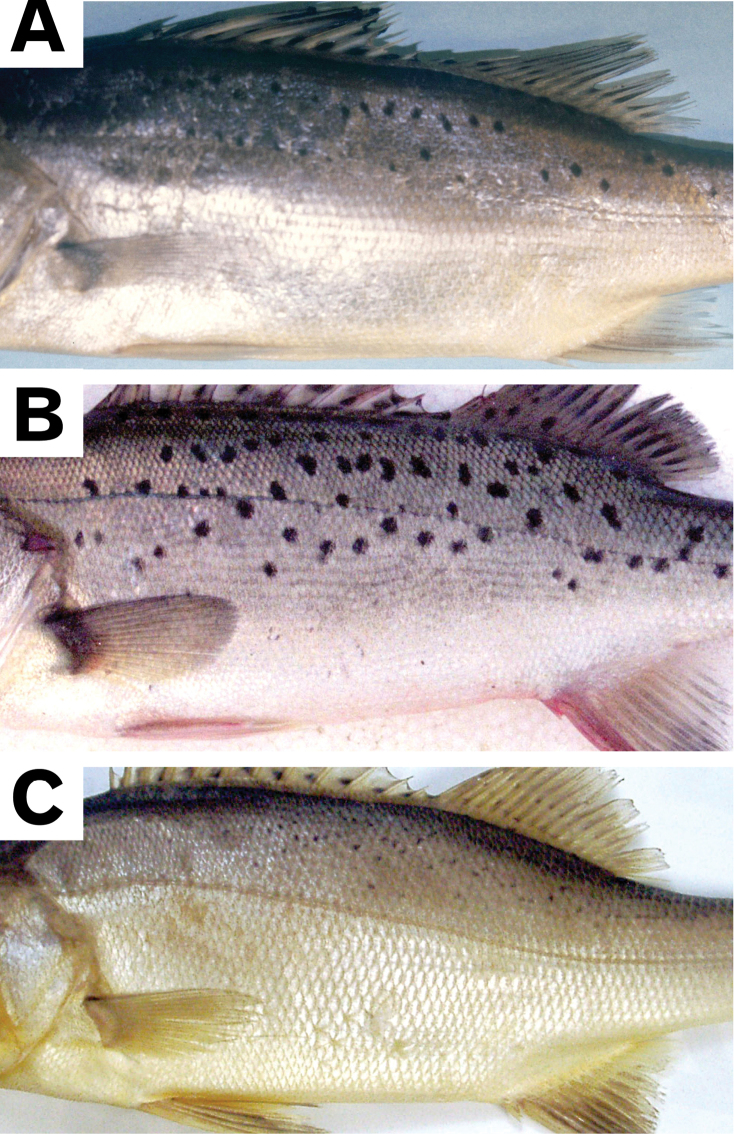
Dots / spots on lateral body regions of *Lateolabraxjaponicus* (**A**), *L.maculatus* (**B**) and *L.latus* (**C**). **A** uncatalogued specimen (168.4 mm SL) **B**BSKU 100773 (254.2 mm SL) **C**KAUM–I. 29117 (219.7 mm SL).

*Lateolabraxmaculatus* has been characterized by many clear black spots on the body, but this character is also problematic as a few individuals of the species lack such spots (Yokogawa and Seki 1995), whereas some individuals of the other two *Lateolabrax* species have dots (Fig. 2). Although Yokogawa and Seki (1995) revealed differences between *L.japonicus* and *L.maculatus* in some meristic characters, including counts of lateral line scales, gill rakers and vertebrae, overlapping ranges between the two species result in no single character separating them completely. Proportional snout length (SNL), also recently used to separate the two species [SNL of *L.maculatus* relatively shorter than in *L.japonicus* (Hatooka 2000, 2013; Yamada et al. 2007)], may also be problematic due to lack of proof of isometric growth. Furthermore, morphology of the first anal pterygiophore (arched and straight in *L.japonicus* and *L.maculatus*, respectively), proposed by Kang (2000), still needs to be validated due to possible growth-related changes.

Thus, morphological identifications of the three *Lateolabrax* species remain problematic, although genetic studies have shown them to be independent species (Yokogawa 1998; Shan et al. 2016). Accordingly, the present study investigated the morphology of the three *Lateolabrax* species in detail, emphasizing growth-related changes, which have received little previous attention, in a search for clear and unequivocal key characters. Concerning this, although the potential of sexual dimorphism is an important issue, *Lateolabrax* species have no reported visual traits to distinguish the gender. Although sex determination requires observations on gonads by dissection, it could not be performed on the catalogued specimens, which represented most of the materials examined in the present study (see Materials and methods), therefore sexual dimorphism was not considered.

## Materials and methods

### Specimens examined

Measurements were based on the following *Lateolabrax* specimens, which have been deposited in the Laboratory of Marine Biology, Faculty of Science, Kochi University (BSKU), Kanagawa Prefectural Museum of Natural History (KPM), the Kagoshima University Museum (KAUM) and Tokushima Prefectural Museum (TKPM), together with some uncatalogued ones. Because presence of some specialized sea bass populations, which resulted from introgressive hybridization between *Lateolabraxjaponicus* and *L.maculatus*, have been reported from Japan (Ariake and Yatsushiro Seas) (Yokogawa et al. 1997; Yokogawa 2002, 2004; Nakayama 2002; Han et al. 2015) and Korea (Yokogawa 2004; Bae et al. 2017), specimens from such areas were not adopted. Further, most specimens of these two species examined in the present study had been previously genetically recognized to be from the pure strains, using isozyme analysis (Yokogawa and Seki 1995).

*Lateolabraxjaponicus* (229 specimens). BSKU 100789–100804 (16), 100826, KPM-NI 9697, 9698, KAUM–I. 82683–82703 (21), 93431–93439 (9), uncatalogued specimens (54) – all Kagawa Pref.; BSKU 101505–101541 (37), Hyogo Pref., Seto Inland Sea; BSKU 100739–100769 (31), 100788, Yamaguchi Pref., Seto Inland Sea; BSKU 66400, KPM-NI 9699 – both Uwajima, Ehime Pref., TKPM-P 352 (20), Tokushima Pref.; KPM-NI 27449, Mie Pref.; KPM-NI 30671, Sagami Bay; BSKU 100837, 100839, 100842, 100845, 100846, 100852, 100854, 100855, 100859–100862 (4), 100865, 100867, 100873, 100874, 100876, 100878, 100879, 100882, 100883, 100886, 100888, 100891, 100893, 100897, 100898, 100900–100902 (3), 100904, 100906, 100907 – all Ishikawa Pref.

*Lateolabraxmaculatus* (170 specimens). BSKU 100770–100787 (18), 101787–101826 (40), a wild strain imported from Yantai, China and cultured in Kagawa, Japan; TKPM-P 1655 (40), uncatalogued specimens (33), a wild strain imported from China (locality unknown) as aquacultural seeds; BSKU 66398, 66399, 66401–66406 (6), TKPM-P 6114, 6140, KPM-NI 9686–9689 (4), 9691–9694 (4), uncatalogued specimens (17) – all Uwajima, Ehime Pref. (presumed escapees from nurseries); TKPM-P 16897, KPM-NI 9696, uncatalogued specimens (2) – all eastern Seto Inland Sea (presumed escapees from nurseries).

*Lateolabraxlatus* (136 specimens). BSKU 101827, Awaji I., Seto Inland Sea; BSKU 100553, 100554, 100556–100561 (6), 101835, TKPM-P 372 – all Tokushima Pref.; KAUM–I. 1895 (4) locality unknown; KAUM–I. 25203, 29117, KPM-NI 24246–24248 (3), 24252–24256 (5), 24648–24656 (9), 24935–24940 (6) – all Yakushima I.; KAUM–I. 33778, Ikarajima I., Yatsushiro Sea.; KAUM–I. 39049–39051 (3), 39055–39058 (4), 39128, 39129, 61956, 64737, 64738, 66393, 66394, 67090, Tanegashima I.; KAUM–I. 42043, 42044, 51058–51068 (11), 54112, 54668, 57963, 58161, 58162, 61406, 61407, 61577, 63162–63169 (8), 63625, 65483–65485 (3), 65671, 80441–80444 (4), Kagoshima Pref. (mainland); KAUM–I. 66081, 75375, 75660, 75815, 75816, Nagasaki Pref.; KPM-NI 21869, 22433, 23429, Shizuoka Pref.; KPM-NI 24566, 24579, 24615, 35333, Miyazaki Pref.; KPM-NI 26185, 26186, 26992, 28599 (3), 29040, Chiba Pref.; KPM-NI 26973, 26975–26979 (5), 26988–26991 (4), Uwajima, Ehime Pref.; KPM-NI 29041–29048 (8), 31568, Kochi Pref.; KPM-NI 29279, 37509, 37919, 37920, Kanagawa Pref.

### Morphological measurements

**Figure 3.**
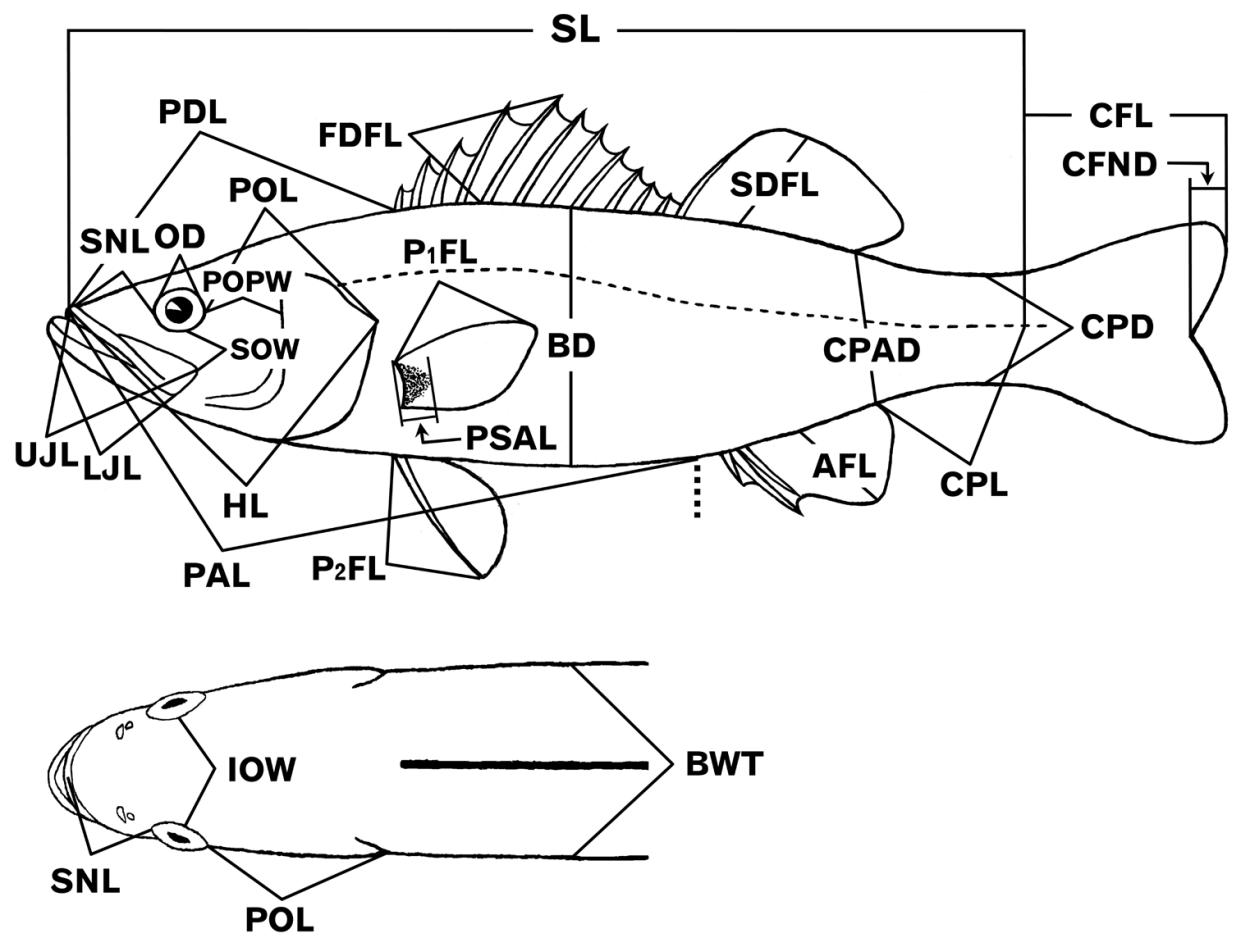
Illustrations of *Lateolabrax* body measurements taken. For abbreviations, see Table 1.

Methods of measurements and counts followed Hubbs and Lagler (1970). Dimensions were taken with calipers (minimum scale 0.1 mm), with particular care for smaller specimens due to the effect of even a small error on the calculated proportion. The characters examined are listed with abbreviations in Table 1 and illustrated in Figure 3. New or uncommon length-measured characters included: post-orbital preopercular width (horizontal distance from orbit posterior edge to preopercle posterior margin), post-orbital length (distance from orbit posterior edge to opercle posterior angle), caudal peduncle anterior depth (distance between posterior ends of dorsal and anal fin bases), caudal fin notch depth (horizontal distance from bottom of notch to margin of naturally spread fin) and pectoral scaly area length (defined by Yokogawa and Seki 1995) (see Figure 3).

**Table 1.** Characters considered for the analysis.

	**Abbreviation**		**Abbreviation**
**Length-measured body characters**		Post-orbital preopercular width	POPW
Standard length	SL	Upper jaw length	UJL
Pre-anus length	PAL	Lower jaw length	LJL
Body depth	BD	**Meristic characters**	
Body width	BWT	Dorsal fin spine	DFS
Caudal peduncle depth	CPD	Dorsal fin soft ray	DFR
Caudal peduncle anterior depth	CPAD	Anal fin spine	AFS
Caudal peduncle length	CPL	Anal fin ray	AFR
Pre-dorsal length	PDL	Pectoral fin ray	P_1_FR
First dorsal fin (longest spine) length	FDFL	Pelvic fin spine	P_2_FS
Second dorsal fin (longest ray) length	SDFL	Pelvic fin ray	P_2_FR
Caudal fin length	CFL	Pored scale on lateral line	LLS
Caudal fin notch depth	CFND	Scale above lateral line	SAL
Anal fin (longest ray) length	AFL	Scale below lateral line	SBL
Pectoral fin length	P_1_FL	Upper-limb gill raker	UGR
Pelvic fin length	P_2_FL	Lower-limb gill raker	LGR
Pectoral scaly area length	PSAL	Total gill raker	TGR
Head length	HL	Abdominal vertebra	AV
**Length-measured cephalic characters**		Caudal vertebra	CV
Snout length	SNL	Total vertebra	TV
Orbital diameter	OD	**Others**	
Inter-orbital width	IOW	Dorsocephalic scale row	DSR
Sub-orbital width	SOW	Ventromandibular scale row	VSR
Post-orbital length	POL	First anal pterygiophore	FAP

Scale row and paired fin ray counts were made on the left side of the body, whereas gill rakers were counted on the first gill arch on the right side by separating the upper and lower limbs of the gill arch. Because counts of pelvic fin-spine (P_2_FS) and soft rays (P_2_FRs) showed no variation (P_2_FS: 1, P_2_FRs: 5 in all specimens), these counts were omitted from the statistical analyses. Abdominal and caudal vertebrae were counted, and first anal fin pterygiophore morphology observed from radiographs.

Total numbers of recognizable black or faint spots / dots on the left side of the body and mid-dorsal aspect of the caudal peduncle (Fig. 2) were counted. Dorsal head squamation [reported as differing between *L.japonicus* and *L.maculatus* (Yokogawa and Seki 1995)], was examined in all three species. Ventromandibular scale rows were also examined on the left side by separating the anterior and posterior parts following Murase et al. (2012), and their status recorded as present, vestigial or absent.

### Statistical computations

For a length-measured dimension (LD), a growth-related proportional change pattern is given by the relationship between base dimension [e.g., standard length (SL) or head length (HL)] and the LD proportion (LD / SL or LD / HL). Because the relationship between SL (or HL) and LD is generally expressed by a power regression formula (LD = *a* SL *^b^*) (allometric growth), the following formula was used (LD / SL = *a* SL *^b^*^-1^). Accordingly, power regressions were applied for the relationships between SL (or HL) and the LD proportions (Table 2).

**Table 2.** Regression parameters and correlation between standard length (SL) or head length (HL) and proportions of length-measured dimensions (LD) [SL = *a* (LD/SL)*^b^*, HL = *a* (LD/HL)*^b^*] of three *Lateolabrax* species.

**Regression**	*** Lateolabrax japonicus ***			*** Lateolabrax maculatus ***			*** Lateolabrax latus ***		
	***a***	***b***	***r***	***a***	***b***	***r***	***a***	***b***	***r***
SL–PAL/SL	64.42	0.004	0.092	74.89	-0.026	-0.524	63.90	0.008	0.270
SL–BD/SL	44.23	-0.108	-0.735	29.94	-0.029	-0.379	33.03	-0.021	-0.240
SL–BWT/SL	8.78	0.075	0.471	10.71	0.048	0.455	8.43	0.079	0.466
SL–CPD/SL	16.55	-0.100	-0.749	11.48	-0.025	-0.353	11.32	0.002	0.034
SL–CPL/SL	22.33	-0.007	-0.069	19.83	0.019	0.216	21.55	-0.010	-0.115
SL–CPAD/SL	21.12	-0.091	-0.686	14.36	-0.014	-0.220	15.05	0.009	0.140
SL–PDL/SL	44.01	-0.041	-0.574	39.76	-0.029	-0.513	45.07	-0.039	-0.711
SL–FDFL/SL	22.72	-0.081	-0.407	12.40	0.008	0.065	22.22	-0.086	-0.541
SL–SDFL/SL	36.65	-0.201	-0.762	17.05	-0.068	-0.443	23.31	-0.091	-0.485
SL–CFL/SL	32.62	-0.085	-0.472	17.40	0.008	0.056	28.45	-0.055	-0.445
SL–CFND/SL	9.30	-0.115	-0.220	2.87	0.077	0.176	25.10	-0.296	-0.781
SL–AFL/SL	28.14	-0.142	-0.713	18.56	-0.061	-0.474	24.60	-0.096	-0.553
SL–P_1_FL/SL	25.19	-0.070	-0.581	16.98	-0.010	-0.109	19.79	-0.024	-0.270
SL–P_2_FL/SL	31.24	-0.101	-0.701	25.47	-0.073	-0.682	23.84	-0.040	-0.357
SL–HL/SL	42.88	-0.054	-0.677	38.39	-0.036	-0.629	46.25	-0.066	-0.836
SL–SNL/SL	8.23	0.002	0.047	11.42	-0.087	-0.664	10.91	-0.027	-0.456
SL–OD/SL	65.54	-0.431	-0.958	42.67	-0.364	-0.945	55.60	-0.368	-0.963
SL–IOW/SL	7.55	-0.020	-0.173	9.31	-0.064	-0.601	7.75	-0.010	-0.082
SL–SOW/SL	2.26	0.067	0.232	1.80	0.135	0.513	2.04	0.070	0.246
SL–POPW/SL	5.47	0.045	0.423	13.03	-0.094	-0.741	7.21	-0.008	0.066
SL–POL/SL	15.94	0.016	0.170	13.46	0.060	0.691	19.07	-0.027	-0.373
SL–UJL/SL	19.09	-0.061	-0.706	20.81	-0.083	-0.778	22.01	-0.071	-0.778
SL–LJL/SL	20.51	-0.058	-0.700	22.29	-0.084	-0.782	21.66	-0.052	-0.629
SL–PSAL/SL^1^							8.14	-0.130	-0.203
SL–POPW/SNL	71.07	0.030	0.314	90.56	0.031	0.222	65.79	0.020	0.149
HL–SNL/HL	20.42	0.057	0.530	28.48	-0.054	-0.453	24.50	0.040	0.533
HL–OD/HL	109.60	-0.400	-0.946	79.68	-0.338	-0.945	93.74	-0.323	-0.950
HL–IOW/HL	18.38	0.033	0.246	23.83	-0.031	-0.292	17.72	0.057	0.359
HL–SOW/HL	5.90	0.127	0.397	5.55	0.178	0.625	4.98	0.143	0.432
HL–POPW/HL	14.81	0.090	0.690	26.66	-0.022	-0.240	16.41	0.061	0.418
HL–POL/HL	39.87	0.073	0.729	38.83	0.099	0.873	42.78	0.041	0.498
HL–UJL/HL	44.57	-0.009	-0.139	51.77	-0.049	-0.667	47.53	-0.006	-0.109
HL–LJL/HL	48.01	-0.005	-0.092	55.24	-0.049	-0.713	47.36	0.014	0.237

^1^ Simple patterned regressions could not be applied for complicated fluctuations in L.japonicus and L.maculatus.

Characteristics that changed with growth were evaluated so as to determine if the changes were isometric or allometric, i.e., regressions between SL (or HL) and LD were transformed into natural logarithms (ln) (lnLD = *a* lnSL + *b*), and a *t* test was used to examine slope significance for the null hypothesis (*a* = 1), according to Zar (2010). When *a* differed significantly from 1, the character was considered to have changed allometrically, i.e., its proportion had increased or decreased with growth. Meristic counts (MC) were regressed using SL (MC = *a* SL + *b*), and a *t* test used to examine slope significance for the null hypothesis (*a* = 0) (Zar 2010). When *a* differed significantly from 0, the character was considered to have changed with growth. In addition, standard errors, which indicated data variation from the regression lines, were calculated during the above analyses (Zar 2010).

To examine inter-specific differences in length-measured characters, regressions between SL (or HL) and LD were also logarithm-transformed (lnLD = *a* lnSL + *b*), since most characters showed allometric growth (Table 3). Parameters of the regressions (*a* and *b*) were compared by analysis of covariance (ANCOVA) (*t* test), following the methods of Yamada and Kitada (2004).

**Table 3.** Regression parameters (slope and intercept) and correlation between logarithm-transformed length-measured characters, together with results of *t* tests to examine significance of slopes for three *Lateolabrax* species (null hypothesis, slope = 1).

**Regression**	*** Lateolabrax japonicus ***			*** Lateolabrax maculatus ***			*** Lateolabrax latus ***		
	**Slope**	**Intercept**	***t***	**Slope**	**Intercept**	***t***	**Slope**	**Intercept**	***t***
ln SL–ln PAL	1.004	-0.44	1.39	0.974	-0.29	-7.97***	1.008	-0.45	3.25*
ln SL–ln BD	0.892	-0.82	-16.35***	0.971	-1.21	-5.31***	0.979	-1.11	-2.87*
ln SL–ln BWT	1.075	-2.43	8.05***	1.048	-2.23	6.62***	1.079	-2.47	6.10***
ln SL–ln CPD	0.900	-1.80	-17.04***	0.975	-2.16	-4.89***	1.002	-2.18	0.40
ln SL–ln CPL	0.993	-1.50	-1.05	1.019	-1.62	2.86*	0.990	-1.53	-1.33
ln SL–ln CPAD	0.909	-1.55	-14.28***	0.986	-1.94	-2.92*	1.009	-1.89	1.63
ln SL–ln PDL	0.959	-0.82	-10.56***	0.971	-0.92	-7.71***	0.961	-0.80	-11.72***
ln SL–ln FDFL	0.919	-1.48	-6.72***	1.008	-2.09	0.85	0.914	-1.50	-7.45***
ln SL–ln SDFL	0.794	-0.97	-17.15***	0.932	-1.77	-6.31***	0.909	-1.46	-6.42***
ln SL–ln CFL	0.914	-1.11	-7.84***	1.008	-1.75	0.70	0.974	-1.35	-2.55
ln SL–ln CFND	0.880	-2.35	-3.41**	1.077	-3.55	2.22	0.704	-1.38	-13.88***
ln SL–ln AFL	0.858	-1.27	-15.17***	0.939	-1.68	-6.97***	0.904	-1.40	-7.67***
ln SL–ln P_1_FL	0.930	-1.38	-10.73***	0.990	-1.77	-1.41	0.976	-1.62	-3.25*
ln SL–ln P_2_FL	0.899	-1.16	-14.81***	0.927	-1.37	-12.06***	0.960	-1.43	-4.42***
ln SL–ln HL	0.946	-0.85	-13.87***	0.964	-0.96	-10.46***	0.934	-0.77	-17.67***
ln SL–ln SNL	1.002	-2.50	0.67	0.913	-2.17	-11.57***	0.973	-2.22	-5.94***
ln SL–ln OD	0.569	-0.42	-50.25***	0.636	-0.85	-37.39***	0.632	-0.59	-41.41***
ln SL–ln IOW	0.980	-2.58	-2.64	0.936	-2.37	-9.71***	0.990	-2.56	-0.95
ln SL–ln SOW	1.067	-3.79	3.60**	1.135	-4.02	7.73***	1.070	-3.89	2.94*
ln SL–ln POPW	1.033	-2.84	5.68***	0.943	-2.26	-7.72***	0.993	-2.63	-0.69
ln SL–ln POL	1.014	-1.82	2.10	1.060	-2.00	12.25***	0.974	-1.66	-4.56***
ln SL–ln UJL	0.939	-1.66	-15.04***	0.917	-1.57	-16.02***	0.929	-1.51	-14.34***
ln SL–ln LJL	0.942	-1.58	-14.74***	0.916	-1.50	-16.11***	0.948	-1.53	-9.34***
ln SNL–ln POPW	1.026	-0.26	4.37***	1.020	0.01	1.71	1.017	-0.36	4.19***
ln HL–ln SNL	1.057	-1.59	9.41***	0.946	-1.26	-6.65***	1.040	-1.41	7.28***
ln HL–ln OD	0.600	0.09	-44.06***	0.662	-0.23	-37.38***	0.677	-0.06	-35.28***
ln HL–ln IOW	1.033	-1.69	3.82**	0.969	-1.43	-3.94**	1.057	-1.73	4.45***
ln HL–ln SOW	1.127	-2.83	6.52***	1.178	-2.89	10.36***	1.143	-3.00	5.55***
ln HL–ln POPW	1.090	-1.91	14.36***	0.978	-1.32	-3.19*	1.061	-1.81	5.32***
ln HL–ln POL	1.073	-0.92	15.93***	1.099	-0.95	23.15***	1.041	-0.85	6.62***
ln HL–ln UJL	0.991	-0.81	-2.11	0.951	-0.66	-11.57***	0.994	-0.74	-1.27
ln HL–ln LJL	0.995	-0.73	-0.19	0.952	-0.59	-13.19***	1.014	-0.75	2.81*

Asterisks indicate significance of t values; single, double and triple asterisks indicate 5%, 1% and 0.1% levels, respectively, after Holm-Bonferroni correction by species.

Because some meristic counts tended to increase significantly with growth (Table 4), they were compared using the Mann-Whitney *U* test (Iwasaki 2006). Example numbers for the *U* test being >20 for all species, *z* values (instead of *U* values) for the normal distribution were calculated after correction for distribution continuity, following Iwasaki (2006).

**Table 4.** Regression parameters (slope and intercept) and correlation between standard length (SL) and meristic counts of three *Lateolabrax* species (null hypothesis, slope = 0).

**Regression**	**Slope**	**Intercept**	***r***	***t***
*** Lateolabrax japonicus ***				
SL–DFS counts	-0.00008	12.87	-0.019	-0.28
SL–DFR counts	-0.00081	13.13	-0.130	-2.05
SL–AFR counts	0.00048	7.56	0.089	1.34
SL–P_1_FR counts	-0.00047	16.96	-0.086	-1.30
SL–LLS counts	0.01207	77.01	0.343	5.50***
SL–SAL counts	-0.00258	15.84	-0.258	-3.98**
SL–SBL counts	0.00057	18.57	0.046	0.68
SL–UGR counts	0.00111	8.63	0.126	1.90
SL–LGR counts	-0.00025	17.93	-0.027	-0.41
SL–TGR counts	0.00086	26.56	0.073	1.10
SL–AV counts	0.00017	16.00	0.073	0.93
SL–CV counts	-0.00068	20.02	-0.108	-1.38
SL–TV counts	-0.00051	36.02	-0.083	-1.80
SL–Dot counts	-0.02297	12.69	-0.198	-2.90*
*** Lateolabrax maculatus ***				
SL–DFS counts	-0.00046	12.95	-0.153	-2.00
SL–DFR counts	-0.00028	13.03	-0.066	-0.86
SL–AFS counts	0.00008	2.98	0.104	1.36
SL–AFR counts	0.00097	7.34	0.217	2.88
SL–P_1_FR counts	0.00079	16.33	0.190	2.50
SL–LLS counts	0.00261	73.45	0.099	1.30
SL–SAL counts	0.00008	15.52	0.009	0.24
SL–SBL counts	0.00477	18.17	0.409	5.72***
SL–UGR counts	0.00139	6.40	0.173	2.24
SL–LGR counts	0.00330	14.70	0.507	7.49***
SL–TGR counts	0.00469	21.11	0.408	5.68***
SL–AV counts	-0.00026	15.97	-0.135	-1.67
SL–CV counts	0.00022	19.00	0.089	1.09
SL–TV counts	0.00003	34.97	-0.012	-0.02
SL–Spot counts	0.02333	33.89	0.126	1.62
*** Lateolabrax latus ***				
SL–DFS counts	-0.00026	13.05	-0.092	-1.08
SL–DFR counts	-0.00041	15.11	0.011	-1.20
SL–AFS counts	-0.00002	3.00	0.001	-0.34
SL–AFR counts	0.00026	9.06	0.002	0.55
SL–P_1_FR counts	-0.00026	16.20	0.004	-0.73
SL–LLS counts	0.00264	72.91	0.169	1.99
SL–SAL counts	-0.00063	13.86	-0.079	-0.92
SL–SBL counts	-0.00013	15.79	-0.014	-0.16
SL–UGR counts	-0.00045	6.83	-0.072	-0.83
SL–LGR counts	-0.00109	17.11	-0.176	-2.07
SL–TGR counts	-0.00154	23.94	-0.166	-1.95
SL–AV counts	0.00004	16.03	0.018	0.22
SL–CV counts	-0.00005	19.92	-0.014	-0.17
SL–TV counts	0.00001	35.95	-0.004	-0.05
SL–Dot counts	-0.06278	24.74	-0.365	-4.53***

Asterisks indicate significance of t values; single, double and triple asterisks indicate 5%, 1% and 0.1% levels, respectively, after Holm-Bonferroni correction by species.

In the above statistical inferences, due to multiple tests being applied simultaneously in each case, multiple comparisons were introduced for the *t* test results, risk percentages for the *t* values being corrected according to total test counts, using the Holm-Bonferroni method (Holm 1979).

## Results

### *Growth-related proportional changes*

#### Body characters

**Figure 4.**
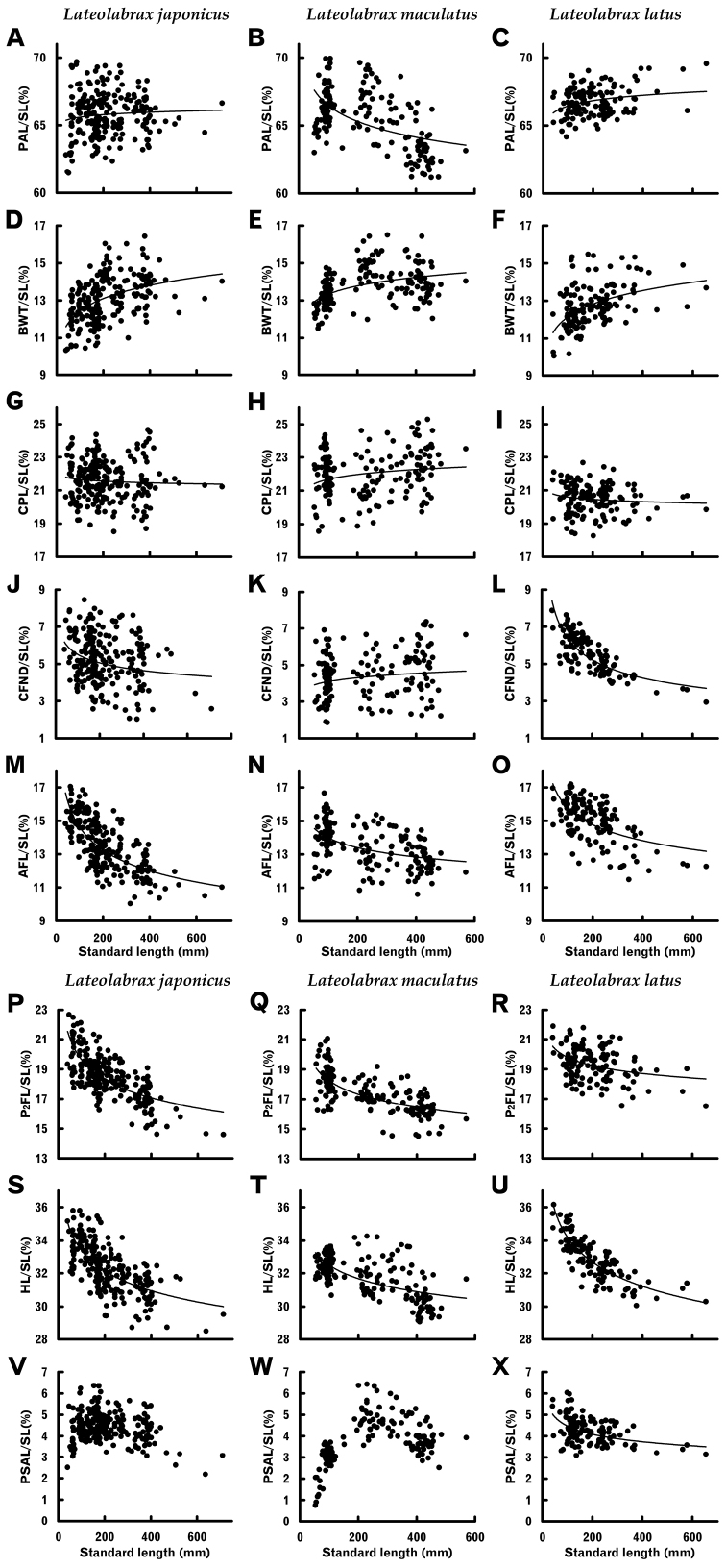
Relationships between standard length and proportions of some length-measured body characters of three *Lateolabrax* species. For character abbreviations, see Figure 3 and Table 1. Solid lines indicate power regression curves (parameters given in Table 2).

**Figure 5.**
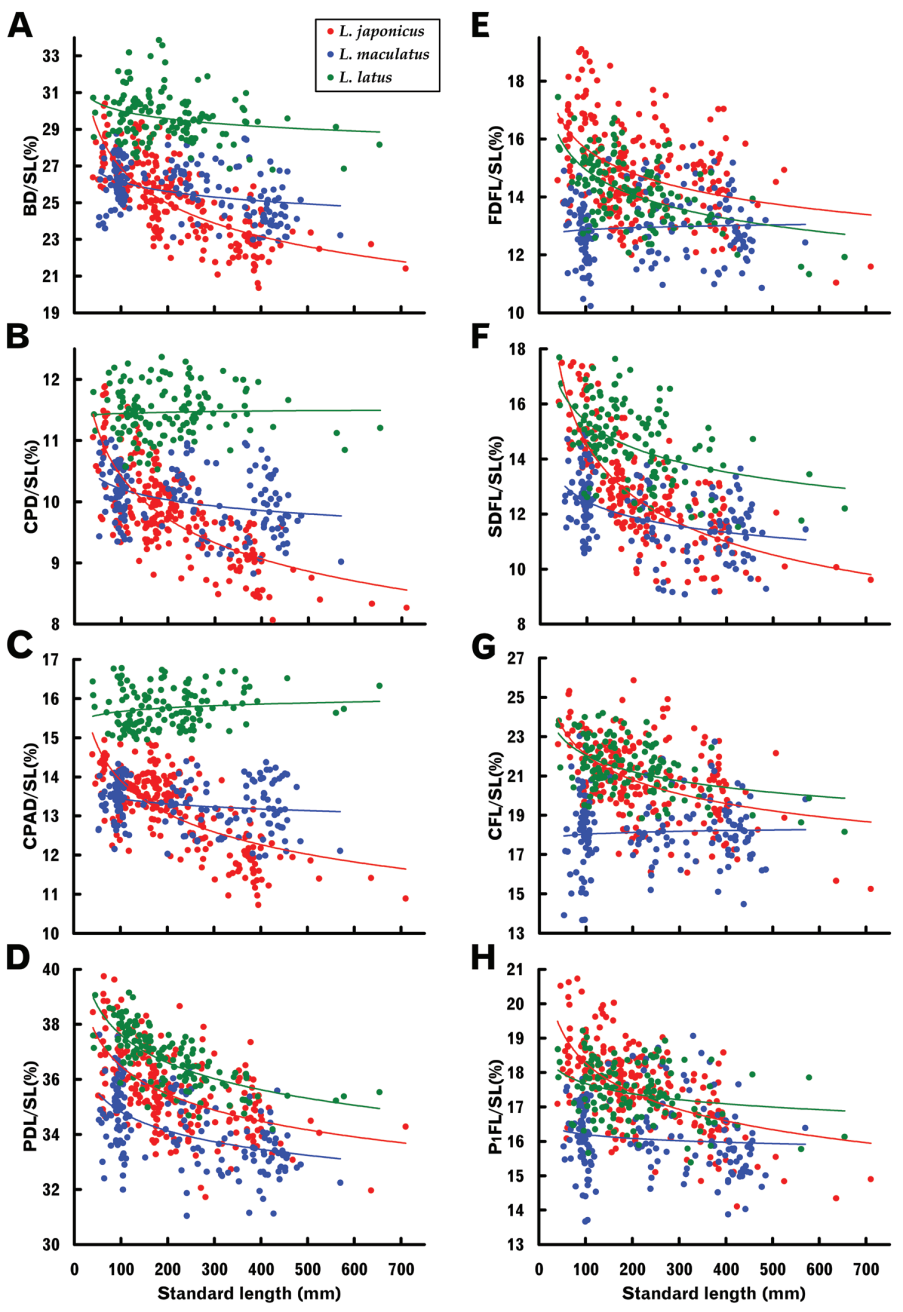
Relationships between standard length and proportions of some length-measured body characters which exhibited prominent plot separation among three *Lateolabrax* species. For character abbreviations, see Figure 3 and Table 1. Solid lines indicate power regression curves (parameters given in Table 2) for each species.

In the three *Lateolabrax* species, slopes of the logarithm-transformed regressions were significantly different from 1 (allometric growth) for most characters (Table 3), indicating that most of the body proportions changed with growth. Relationships between standard length (SL) and length-measured body proportions are shown graphically by species in Figure 4, those with prominent plot separation between species being shown with multiple specific plots in Figure 5.

Similar patterns of growth-related proportional changes common to the three species were observed for some characters, viz., significant positive allometric growth (proportions increased with growth) in body width and significant negative allometric growth (proportions decreased with growth) in head (HL) and pre-dorsal length (PDL), and second dorsal, anal and pelvic fin (longest ray) lengths (SDFL, AFL and P_2_FL), although patterns of the regression curves or plot distributions for the three species sometimes varied from one another (Figs 4, 5, Table 3). Differing specific growth-related proportional changes were evident for some other characters, e.g., pre-anus length (PAL), isometric growth in *L.japonicus*, negative and positive allometric growth in *L.maculatus* and *L.latus*, respectively (Fig. 4A–C, Table 3); and caudal fin notch depth (CFND), modestly and highly negative allometric growth in *L.japonicus* and *L.latus*, respectively, and isometric growth in *L.maculatus* (Fig. 4G–I, Table 3). In the latter, however, despite specific growth-related patterns, ranges of the CFND / SL proportions taken over the entire range of SLs were similar to one another, viz., 2.0–8.4%, 1.9–7.4% and 2.9–7.9%, in *L.japonicus*, *L.maculatus* and *L.latus*, respectively (Fig. 4J–L).

#### Cephalic characters

**Figure 6.**
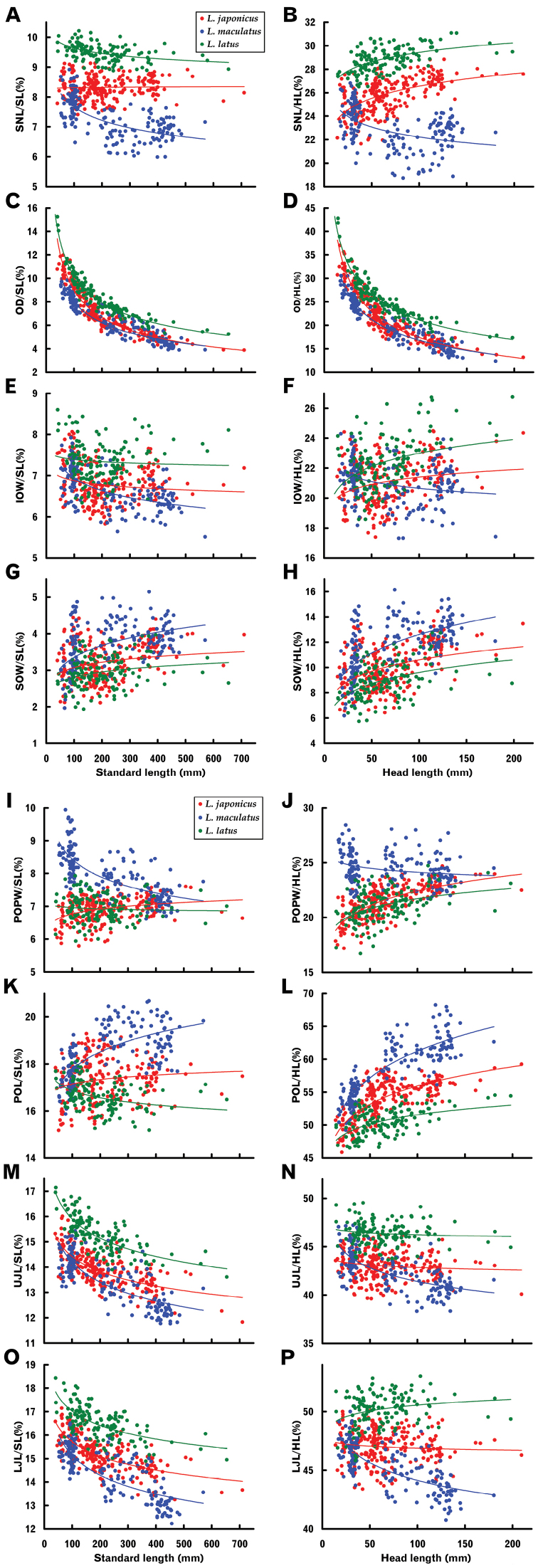
Relationships between standard length or head length and proportions of length-measured cephalic characters of three *Lateolabrax* species. For character abbreviations, see Figure 3 and Table 1. Solid lines indicate power regression curves (parameters given in Table 2) for each species.

For length-measured dimensions (LD) of cephalic characters, SL-based (SL–LD / SL) and HL-based relationships (HL–LD / HL) are illustrated in pairs with multiple specific plots in Figure 6. In each species, significant allometric growth was recognized in most length-measured cephalic characters as well as length-measured body characters (Table 3). In particular, negative allometric growth was so significant for orbital diameter (OD) (very high *t* values, see Table 3) that the plots for each all formed typical arched curves (Fig. 6C, D), indicating rapid decrement of OD proportions with growth. Such acute relative OD decrement in the three species was clearly apparent from photographs (Fig. 1).

Growth-related proportional change patterns based on SL and HL were inconsistent with each other for some characters in *L.japonicus* and *L.latus*, e.g., snout length (SNL) of *L.japonicus* was isometric and positively allometric for SL and HL, respectively; that of *L.latus* was negatively and positively allometric for SL and HL, respectively (Fig. 6A, B, Table 3). While the patterns were consistent between the SL- and HL-based relationships in *L.maculatus* for all cephalic characters (Fig. 6A–P, Table 3), allometric increment / decrement rates varied in the two-way relationships e.g., proportions of post-orbital preopercular width (POPW) decreased with growth acutely and slightly for SL and HL, respectively (Fig. 6I, J, Table 3).

As well as some body characters, specific proportional change patterns were recognized for some characters, e.g., SL-based relationships of POPW, exhibiting isometric growth in *L.japonicus*, and positive and negative allometric growth in *L.maculatus* and *L.latus*, respectively (Fig. 6K, Table 3); and SNL, exhibiting isometric growth in *L.japonicus*, and high and modest negative allometric growth in *L.maculatus* and *L.latus*, respectively (Fig. 6A, Table 3).

#### Pectoral scaly area length

**Figure 7.**
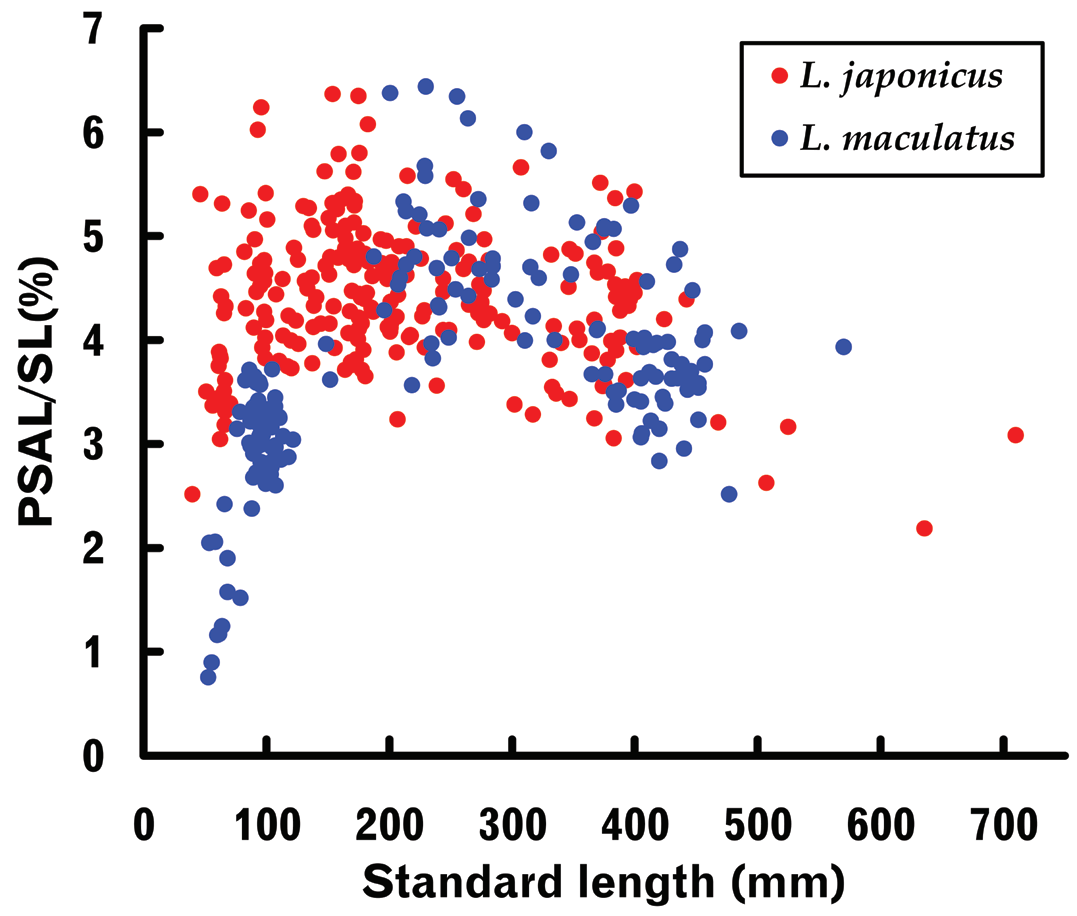
Relationships between standard length (SL) and pectoral scaly area length (PSAL) proportions for *Lateolabraxjaponicus* and *L.maculatus*.

The relationship between SL and pectoral scaly area length (PSAL) in *L.latus* was well fitted to a power regression (like many other body and cephalic length-measured characters), the PSAL / SL proportion gradually decreasing with growth (Fig. 4X, Table 2). In the other two species, however, proportional PSAL rapidly increased from the smallest specimens to a peak and thereafter gradually decreased (Fig. 4V, W), therefore being unsuitable for simple patterned regressions. Synchronous plotting for the two species showed the proportional PSAL of *L.maculatus* to be distinctly less than that of *L.japonicus* during the initial stage (< ca. 150 mm SL), although plots of the two species largely overlapped during the subsequent decreasing stage (Fig. 7). The proportional PSAL of *L.latus* during the former stage was much greater than in the other two species (Fig. 4V–X).

### *Inter-specific differences*

#### Length-measured body and cephalic characters

Plot separation of *L.latus* from the other two species was prominent for vertical body dimensions of body depth (BD), caudal peduncle depth (CPD) and caudal peduncle anterior depth (CPAD), *L.japonicus* and *L.maculatus* both showing significant negative allometric growth, the degree of relative decrease being especially acute in the former. Although BD of *L.latus* showed slight negative allometric growth, CPD and CPAD were regarded as isometric (Fig. 5A–C, Table 3). However, despite considerable plot separation of BD and CPD between *L.latus* and the other species, plots of the three species overlapped for the smaller size class (< ca. 200 mm SL) (Fig. 5A, B). CPAD plots for *L.latus* were entirely separated from those of the other two species (border level 15%) (Fig. 5C). Although similar plot separation for caudal peduncle length (CPL) in *L.latus* was also apparent, ranges of proportional CPL of the three species almost overlapped due to considerable variation in plot distribution in *L.japonicus* and *L.maculatus* (Fig. 4G–I).

Plot separation of first and second dorsal (FDFL and SDFL), caudal (CFL) and pectoral (P_1_FL) fin lengths was also apparent between *L.japonicus* and *L.maculatus* (Fig. 5E–H), the former showing significant negative allometric growth of each feature, whereas the latter exhibited isometric growth for all, except SDFL (Table 3). Proportions in the former were distinctively greater than in the latter in the smaller size class (< ca. 200 mm SL), although plots of the two species overlapped in the larger size class (> ca. 200 mm SL), since fin length proportions decreased and did not change with growth, respectively (Fig. 5E, G, H). Such proportional differences in fin length in the smaller size class between the two species were clearly apparent from photographs (Fig. 1A, C).

Upward plot separation of *L.latus* from the other two species was prominent for SNL and upper and lower jaw lengths (UJL and LJL), there being almost no overlap with *L.maculatus* and only modest overlap with *L.japonicus* (Fig. 6A, B, M–P). Plots of OD for *L.latus* were similarly upwardly separated from those of the other two species (Fig. 6C, D), especially in the HL-based graph (Fig. 6D). Post-orbital length (POL) plots for *L.latus* were shifted downward from those of the other two species (Fig. 6K, L), plot separation being more prominent in the HL-based graph (Fig. 6L).

On the other hand, plot separation between *L.japonicus* and *L.maculatus* was prominent for SNL, POPW and POL (Fig. 6A, B, I–L). SNL plots for the two species overlapped in the smaller size class (< ca. 200 mm SL), subsequently progressively separating with growth due to the proportional SNL of *L.maculatus* decreasing with growth (negative allometry), to a border level of ca. 7.7% (Fig. 6A) in the larger size class (> ca. 200 mm SL). This phenomenon was more apparent in the HL-based relationship because proportional SNL in *L.japonicus* increased with growth (positive allometry) (Fig. 6B, Table 3), unlike that for the SL-based relationship (isometric growth) (Fig. 6A, Table 3). Similar patterns were observed for POL, plots of the two species overlapping in the smaller size class (< ca. 200 mm SL), but subsequently separating to a certain extent with growth due to a proportional POL increase in *L.maculatus* (positive allometry) (Fig. 6K, L). In contrast, POPW plots of the two species were completely separated from each other in the smaller size class (< ca. 200 mm SL), having a border level of ca. 7.5%, but progressively overlapped with growth due to the proportional POPW of *L.japonicus* and *L.maculatus* increasing and decreasing with growth, respectively (Fig. 6I, J).

**Figure 8.**
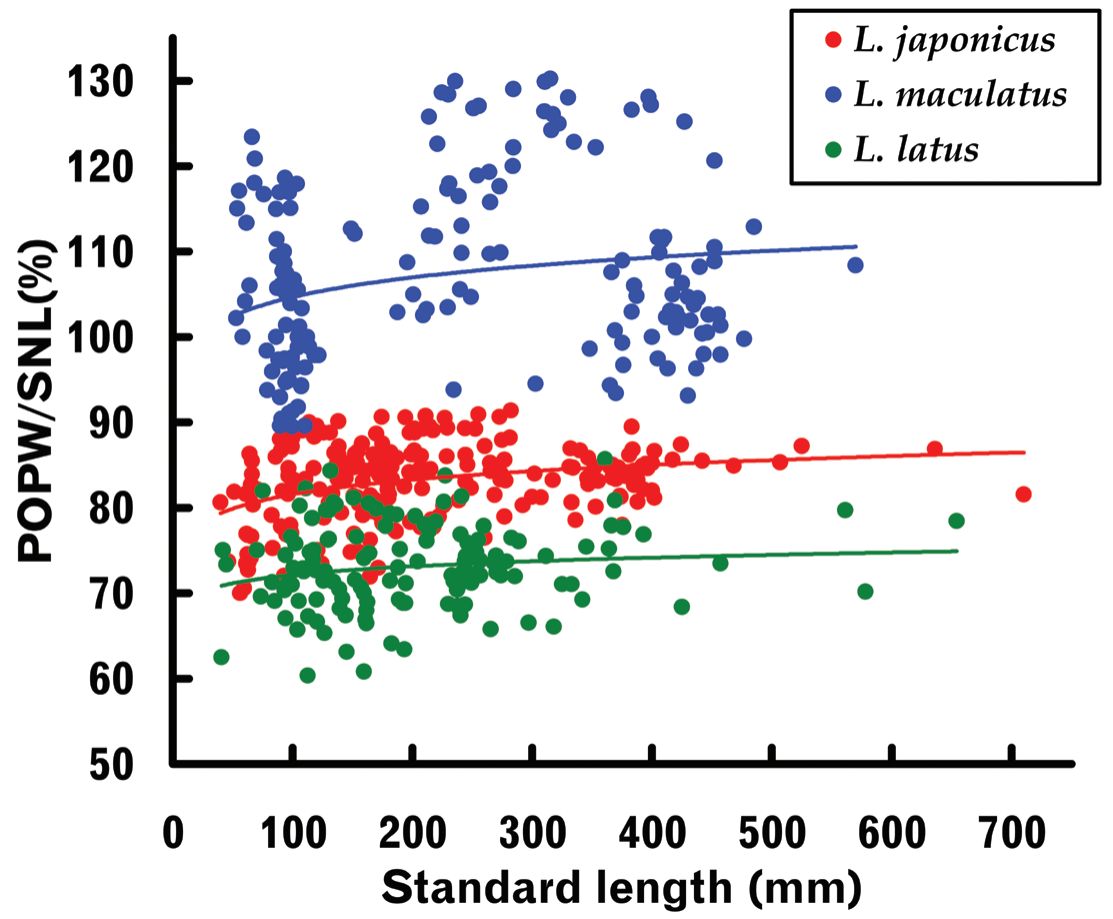
Relationships between standard length and (post-orbital preopercular width) / (snout length) proportions of three *Lateolabrax* species. Solid lines indicate power regression curves (parameters given in Table 2) for each species.

POPW proportional to SNL is shown graphically in Figure 8. The SL–POPW / SNL regressions were positively allometric for *L.japonicus* and *L.latus*, and isometric for *L.maculatus* (Table 3). Plots for *L.japonicus* and *L.maculatus* were separated from each other almost entirely throughout all size ranges (border level 90%), following a slight plot overlap at ca. 100 mm SL (Fig. 8). In addition, plots for *L.latus* were displaced well downward from the other two species, despite some overlap with *L.japonicus* (Fig. 8).

#### Meristic characters

**Figure 9.**
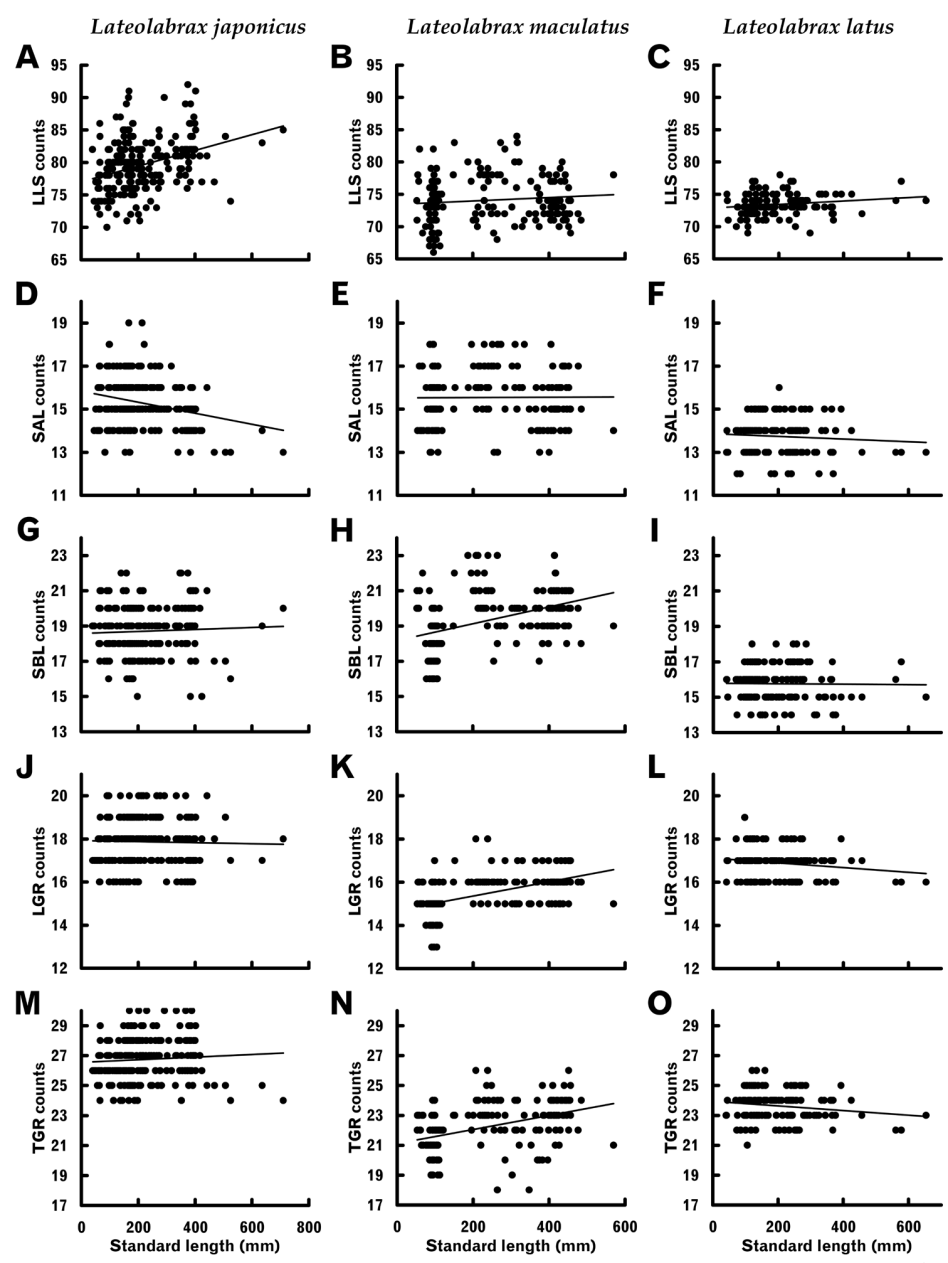
Relationships between standard length and some meristic characters which exhibited growth-related changes in some *Lateolabrax* species. For character abbreviations, see Figure 3 and Table 1. Solid lines indicate linear regressions (parameters given in Table 4).

The *t* tests of regressions between SL and meristic counts (null hypothesis, slope = 0) proved significant for scales on (LLS) and above the lateral line (SAL) in *L.japonicus*, and scales below the lateral line (SBL) and gill raker counts [lower limb and total (LGR and TGR, respectively)] in *L.maculatus* (Table 4). Whereas SAL counts in *L.japonicus* tended to decrease with growth (Fig. 9), having negative slope values (Table 4), the remaining characters tended to increase (Fig. 9, Table 4). No significant differences in any meristic characters were found in *L.latus* (Table 4), indicating that none changed with growth in that species.

**Figure 10.**
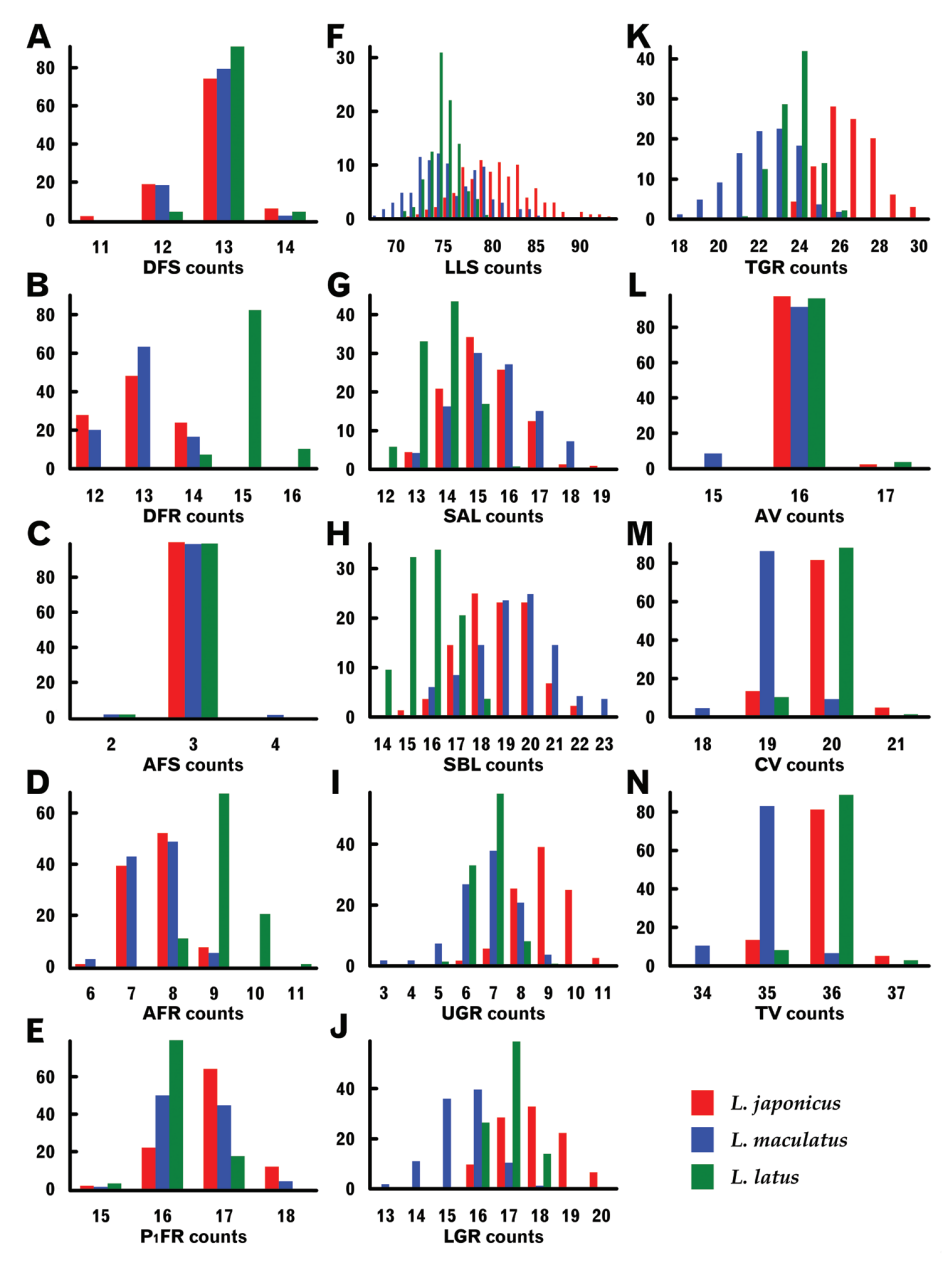
Histograms of meristic characters of three *Lateolabrax* species. For character abbreviations, see Figure 3 and Table 1. Vertical axes indicate frequencies (%).

Figure 10 shows multiple specific frequency histograms for all meristic characters, *L.latus* clearly differing from the other two species in dorsal (DFR) and anal fin ray (AFR) counts (there being only slight range overlaps), as well as in pectoral fin ray (P_1_FR) and SBL counts, again with some range overlaps. Notably, DFRs (14) in *L.latus* had only a 7.4% overlap of the ranges of the other two species, the latter differing significantly in vertebral counts [caudal and total (CV and TV, respectively)] and ranges of LLS, LGR and TGR. However, no species had a meristic character count range that was entirely separated from those of the other species.

##### Spots / dots on lateral body region

**Figure 11.**
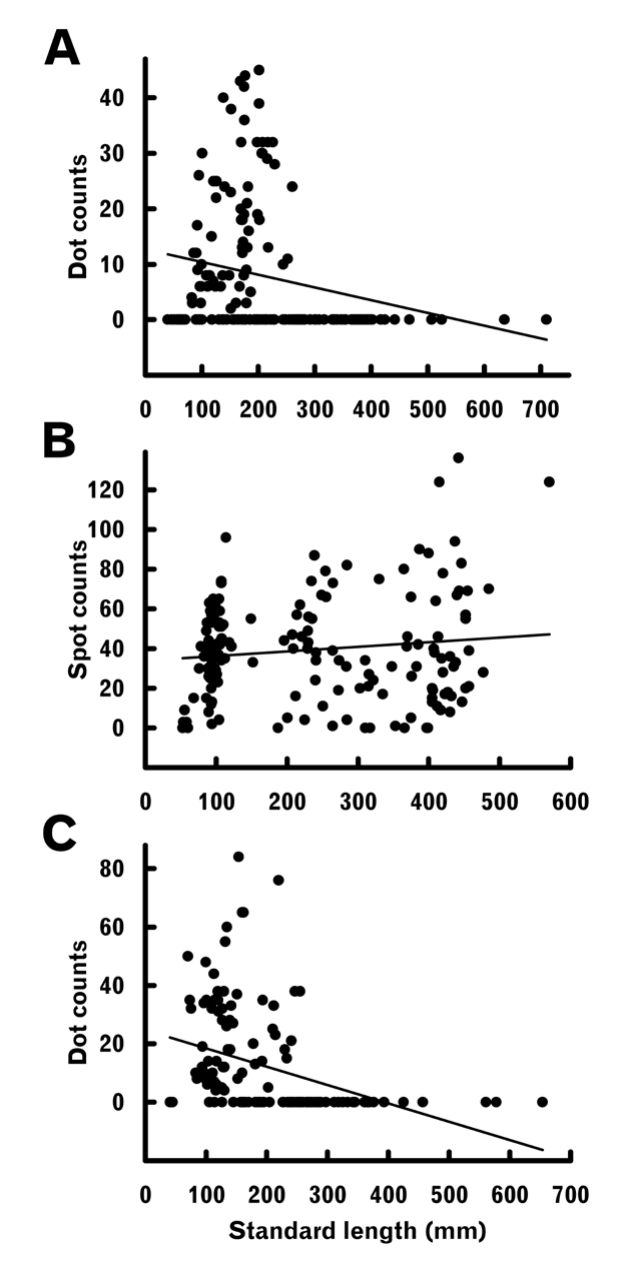
Relationships between standard length and dot / spot counts on lateral body regions of *Lateolabraxjaponicus* (**A**), *L.maculatus* (**B**) and *L.latus* (**C**). Solid lines indicate linear regressions (parameters given in Table 4).

Some examples of *L.japonicus* and *L.latus* had small and fine dots, respectively, on the lateral body region (Fig. 2A, C), whereas *L.maculatus* usually had many clear black spots (Fig. 2B). In both of the former, dots appeared to be limited to some smaller specimens (Fig. 11A, C), the maximum sizes of specimens with dots being 260.6 mm SL (BSKU 100765) and 254.8 mm SL (KAUM–I. 66393), respectively. The *t* tests indicated significant regressions between SL and dot counts for the two species (null hypothesis, slope = 0 rejected), both indicating negative correlations (minus slope values) (Fig. 11A, C, Table 4). The proportions of dotted specimens of the total material examined were 35.6% and 46.3% (51.9 and 60.0% for specimens <250 mm SL) in *L.japonicus* and *L.latus*, respectively. In *L.maculatus*, spot counts were typically abundant (ca. 40 on average), but variable (absent in 4.9% of specimens) (Fig. 11B) and not related to body size, a *t* test (null hypothesis, slope = 0) indicating no significant regression between SL and spot counts (Table 4).

##### Squamation on dorsal head region

**Figure 12.**
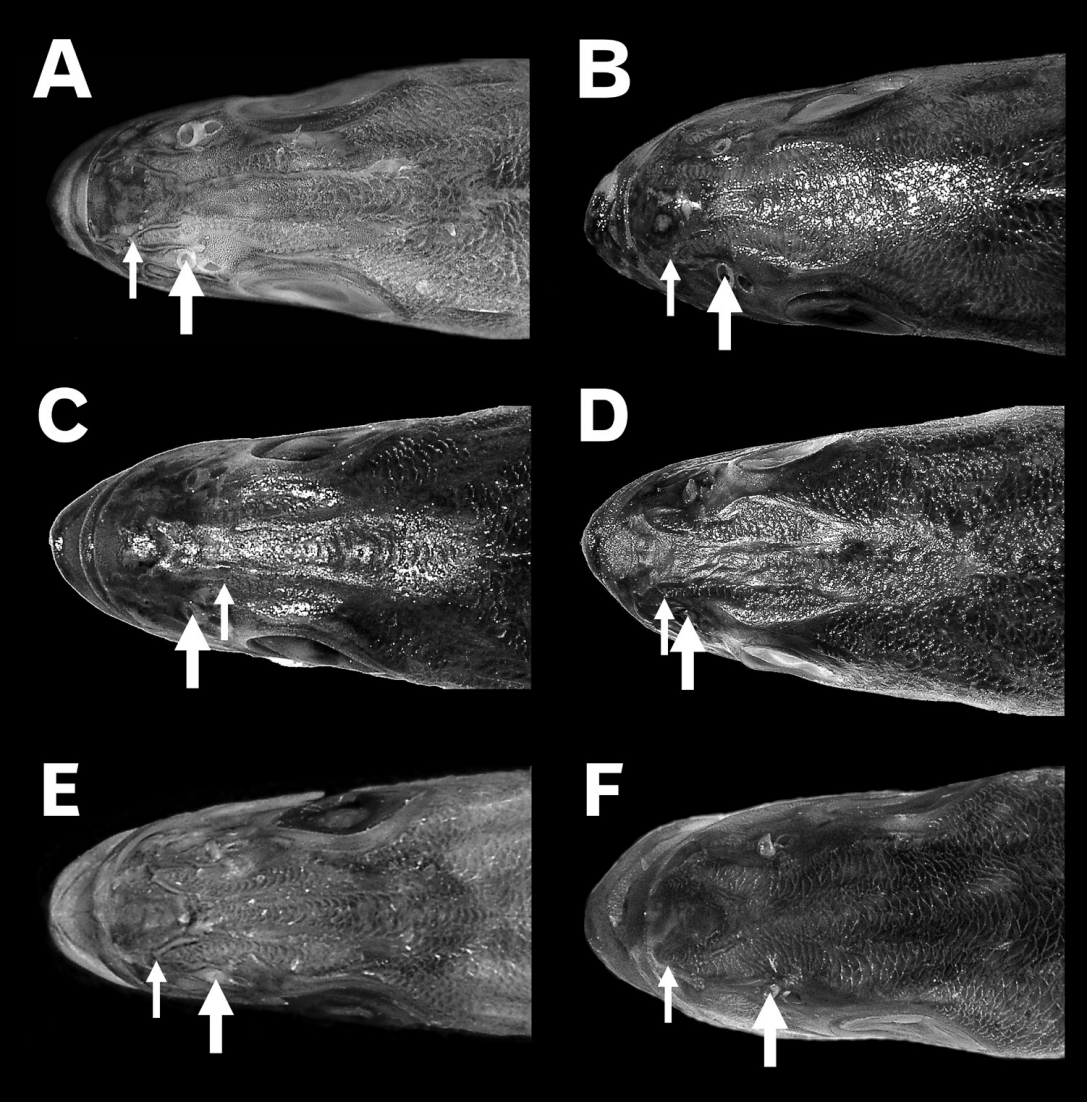
Squamation on dorsal head regions of *Lateolabraxjaponicus* (**A, B**), *L.maculatus* (**C, D**) and *L.latus* (**E, F**). Thick arrows indicate anterior nostrils, thin arrows indicate anterior edges of dorsocephalic scale rows. **A**KAUM–I. 93435 (137.0 mm SL) **B**BSKU 100803 (265.2 mm SL) **C** uncatalogued specimen (104.9 mm SL) **D**BSKU 100773 (254.2 mm SL) **E**KAUM–I. 39058 (114.2 mm SL) **F**KPM-NI 24255 (240.1 mm SL).

Post-juvenile specimens (> ca. 70 mm SL) of the three *Lateolabrax* species had a pair of scale rows (dorsocephalic scale rows, DSRs) extending forward from the inter-orbital area, which was densely covered with fine scales (Fig. 12). DSRs in *L.japonicus* and *L.latus* were well developed distally, with anterior edges always beyond the anterior nostril position (ANP) (Fig. 12A, B, E, F), and almost reaching the upper lip in large specimens of *L.latus* (Fig. 12F). On the other hand, DSRs in small specimens of *L.maculatus* were almost entirely restricted to the inter-orbital region, not extending beyond ANPs (Fig. 12C), although gradual development with growth resulted in DSRs extending beyond the ANP in specimens > ca. 150 mm SL (Fig. 12D).

##### Squamation on ventral head region

Some individuals of the three *Lateolabrax* species had a pair of ventromandibular scale rows (VSRs), VSR status by body size being summarized in Table 5. In *L.japonicus*, although VSRs were entirely absent in specimens ≤ 100 mm SL, a few ca. 150 mm SL had vestigial VSRs. Subsequently, the proportion of specimens with VSRs gradually increased with growth, those lacking anterior and posterior VSRs comprising 25.0% and 0%, respectively, of the largest size class (> 400 mm SL). VSRs were entirely absent in *L.maculatus* specimens < 200 mm SL, appearing in a few just over 200 mm SL. Subsequently, the proportion of specimens with VSRs gradually increased with growth, those without anterior and posterior VSRs comprising 36.4% and 0%, respectively, of the largest size class (> 400 mm SL). Although VSRs were absent in most *L.latus* specimens ≤ 100 mm SL, a few over 90 mm SL had incipient or established VSRs. Subsequently, the proportion of specimens with VSRs rapidly increased with growth, including most up to 300 mm SL and all > 300 mm SL. Notably, 100–200 mm SL specimens with VSRs showed greater development of the anterior portion, contrary to the developmental pattern displayed by the other two species. The prominence of VSR appearance was ranked: 1 *L.latus*, 2 *L.japonicus*, 3 *L.maculatus*.

**Table 5.** Frequencies (%) of ventromandibular scale row status in three Lateolabrax species.

**SL range (mm)**	**Anterior part**			**Posterior part**		
	**Present**	**Vestigial**	**Absent**	**Present**	**Vestigial**	**Absent**
*** Lateolabrax japonicus ***						
≤100	0.0	0.0	100.0	0.0	0.0	100.0
100–200	0.0	14.3	85.7	10.7	21.4	67.9
200–300	5.0	25.0	70.0	35.0	30.0	35.0
300–400	5.3	26.3	68.4	31.6	57.9	10.5
>400	25.0	50.0	25.0	37.5	62.5	0.0
*** Lateolabrax maculatus ***						
≤100	0.0	0.0	100.0	0.0	0.0	100.0
100–200	0.0	0.0	100.0	0.0	0.0	100.0
200–300	0.0	18.2	81.8	22.7	54.5	22.7
300–400	5.6	55.6	38.9	55.6	27.8	16.7
>400	12.1	51.5	36.4	84.8	15.2	0.0
*** Lateolabrax latus ***						
≤100	0.0	13.3	86.7	6.7	13.3	80.0
100–200	70.5	18.0	11.5	49.2	19.7	31.1
200–300	95.1	4.9	0.0	97.6	2.4	0.0
300–400	100.0	0.0	0.0	100.0	0.0	0.0
>400	100.0	0.0	0.0	100.0	0.0	0.0

#### Morphology of first anal pterygiophore

**Figure 13.**
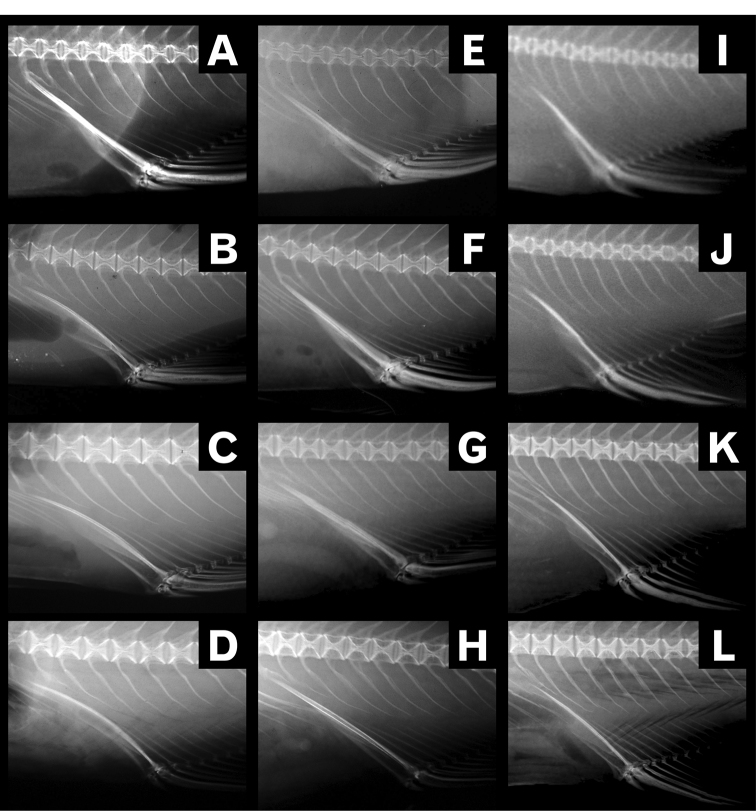
Radiographs of first anal pterygiophores in *Lateolabraxjaponicus* (**A–D**), *L.maculatus* (**E–H**) and *L.latus* (**I–L**), according to body size by species. **A**KAUM–I. 82683 (65.6 mm SL) **B**BSKU 100883 (96.8 mm SL) **C**BSKU 100756 (252.4 mm SL) **D**KPM-NI 9697 (317.0 mm SL) **E** uncatalogued specimen (58.4 mm SL) **F**TKPM-P 1655-6 (95.2 mm SL) **G**BSKU 100771 (250.8 mm SL) **H**KPM-NI 9686 (364.0 mm SL) **I**KAUM–I. 1895-4 (70.3 mm SL) **J**KAUM–I. 64737 (SL 94.2 mm) **K**KPM-NI 24650 (265.4 mm SL) **L**KAUM–I. 57963 (342.0 mm SL).

All three *Lateolabrax* species had a well-developed first anal pterygiophore (FAP), which comprised a short thin plate-like anterior part and a long thick spiny posterior part (Fig. 13). In *L.japonicus*, although the FAPs were straight in small specimens (< ca. 90 mm SL) (Fig. 13A), they became modestly arched in larger specimens (Fig. 13B–D), suggesting a growth-related morphological change. In contrast, the FAPs in *L.maculatus* remained straight (morphologically stable) regardless of body size (Fig. 13E–H). In *L.latus*, on the other hand, although the FAPs were straight in some specimens (Fig. 13I, K), they were slightly arched distally in others (Fig. 13J, L), thus showing neither growth-related morphological change nor morphological stability. As such, relationships between body size and FAP morphology were specifically unique.

#### Statistical differences

Analyses of covariance (ANCOVA) for regressions of logarithm-transformed length-measured characters by pairwise comparisons for the three *Lateolabrax* species indicated significant differences in the slopes or intercepts of all such characters (Table 6). In general, significance (*t* values) between *L.japonicus* and *L.latus*, and *L.maculatus* and *L.latus* were greater than those between *L.japonicus* and *L.maculatus*, suggesting that *L.latus* exhibited greater morphological differences from the other two species (Table 6). High significance levels between the species were apparent for the SNL–POPW relationship (*t* values for intercepts ca. 28–44), in which the scatter plots were almost entirely separated from one another (Fig. 8). The next highest significance levels between the species were for vertical body dimensions (BD, CPD and CPAD), which also exhibited considerable plot separation from one another (Fig. 5) (*t* values of ca. 10 for slopes between *L.japonicus* and *L.maculatus* and between *L.japonicus* and *L.latus* and ca. 27–37 for intercepts between *L.maculatus* and *L.latus*) (Table 6).

**Table 6.** Results of analysis of covariance (ANCOVA) (*t* test) to compare regression parameters of logarithm-transformed length-measured characters between three *Lateolabrax* species.

Regression	*L.japonicus* × *L.maculatus*		*L.japonicus* × *L.latus*		*L.maculatus* × *L.latus*	
	Slope	Intercept	Slope	Intercept	Slope	Intercept
ln SL–ln PAL	7.00***	–	1.03	6.61***	7.08***	–
ln SL–ln BD	9.03***	–	8.16***	–	0.91	26.57***
ln SL–ln BWT	2.34	6.92***	0.22	2.58*	2.22	9.23***
ln SL–ln CPD	9.51***	–	10.97***	–	3.26	26.59***
ln SL–ln CPL	2.81	2.97*	0.29	9.35***	2.69	11.00***
ln SL–ln CPAD	9.41***	–	10.18***	–	2.91	36.84***
ln SL–ln PDL	2.22	11.60***	0.25	10.07***	1.83	21.83***
ln SL–ln FDFL	5.75***	–	0.30	5.13***	5.99***	–
ln SL–ln SDFL	8.52***	–	6.02***	–	1.23	18.51***
ln SL–ln CFL	6.05***	–	3.45*	–	1.86	16.84***
ln SL–ln CFND	3.99**	–	3.49*	–	7.37***	–
ln SL–ln AFL	6.28***	–	2.88	12.25***	2.25	11.23***
ln SL–ln P_1_FL	6.17***	–	4.24**	–	1.26	12.21***
ln SL–ln P_2_FL	3.07	9.89***	5.18***	–	2.96	16.28***
ln SL–ln HL	3.45*	–	1.82	5.42***	5.30***	–
ln SL–ln SNL	9.97***	–	3.68*	–	5.53***	–
ln SL–ln OD	5.26***	–	4.66***	–	0.26	28.99***
ln SL–ln IOW	4.29**		0.73	10.95***	4.27**	–
ln SL–ln SOW	2.64	7.96***	0.08	5.20***	2.15	12.35***
ln SL–ln POPW	10.37***	–	3.61*	–	4.15**	–
ln SL–ln POL	5.43***	–	3.90**	–	10.54***	–
ln SL–ln UJL	3.42*	–	1.44	25.97***	1.55	26.46***
ln SL–ln LJL	4.05**	–	0.79	22.93***	3.76*	–
ln SNL–ln POPW	0.48	33.61***	0.76	27.56***	0.18	44.42***
ln HL–ln SNL	11.07***	–	1.82	23.86***	7.76***	–
ln HL–ln OD	4.84***	–	5.29***	–	1.02	28.82***
ln HL–ln IOW	5.47***	–	1.52	7.78***	5.92***	–
ln HL–ln SOW	1.95	9.36***	0.46	6.42***	1.14	15.08***
ln HL–ln POPW	12.17***	–	2.40	2.74*	6.34***	–
ln HL–ln POL	4.04**	–	4.15**	–	7.64***	–
ln HL–ln UJL	6.89***	–	0.38	22.63***	6.19***	–
ln HL–ln LJL	7.92***	–	2.84	3.37**	9.71***	–

Numbers indicate t values given by ANCOVA. Asterisks indicate significance of t vales; single, double and triple asterisks indicate 5%, 1% and 0.1% levels, respectively, after Holm-Bonferroni correction. Bars indicate that calculation was not demonstrated because significance was recognized for the slope and ANCOVA was therein terminated.

Although the Mann-Whitney *U* tests for pairwise comparisons of meristic characters of the three species found significant differences in many, significance was not apparent for others, including counts of vertical fin rays [dorsal fin spines (DFSs), DFRs and AFRs] between *L.japonicus* and *L.maculatus*, and vertebrae [abdominal vertebrae (AVe), CVe and TVe] between *L.japonicus* and *L.latus* (Table 7).

**Table 7.** Results of the Mann-Whitney *U* test (*z* values) to compare meristic counts between three *Lateolabrax* species.

**Character**	***L.japonicus* × *L.maculatus***	***L.japonicus* × *L.latus***	***L.maculatus* × *L.latus***
DFS counts	0.37	3.00*	3.64**
DFR counts	0.12	16.22***	15.60***
AFS counts	0.00	1.29	0.64
AFR counts	1.39	14.64***	14.11***
P_1_FR counts	5.69***	10.62***	5.77***
LLS counts	11.53***	13.74***	0.89
SAL counts	2.04	11.50***	11.47***
SBL counts	3.57**	14.43***	13.88***
UGR counts	14.31***	14.58***	0.65
LGR counts	15.45***	8.83***	11.76***
TGR counts	16.54***	15.13***	7.81***
AV counts	4.23***	0.64	4.15***
CV counts	13.58***	0.01	13.45***
TV counts	14.82***	0.73	14.09***

Asterisks indicate significance of z vales; single, double and triple asterisks indicate 5%, 1% and 0.1% levels, respectively, after Holm-Bonferroni correction.

Standard errors (SEs) for regression lines between logarithm-transformed SL and length-measured characters, and between SL and meristic characters are summarized in Table 8. For many characters, *L.latus* had the lowest SE values among the three species, followed by *L.japonicus* (Table 8). In general, degrees of SE could be ranked: 1 *L.maculatus*, 2 *L.japonicus*, 3 *L.latus*.

**Table 8.** Standard errors for morphological character regressions of three *Lateolabrax* species.

**Regression**	*** L. japonicus ***	*** L. maculatus ***	*** L. latus ***
ln SL–ln PAL	0.024	0.029	0.015
ln SL–ln BD	0.057	0.050	0.043
ln SL–ln BWT	0.080	0.064	0.077
ln SL–ln CPD	0.051	0.046	0.036
ln SL–ln CPL	0.055	0.060	0.044
ln SL–ln CPAD	0.055	0.044	0.031
ln SL–ln PDL	0.033	0.033	0.020
ln SL–ln FDFL	0.103	0.084	0.068
ln SL–ln SDFL	0.094	0.096	0.084
ln SL–ln CFL	0.085	0.095	0.068
ln SL–ln CFND	0.273	0.299	0.119
ln SL–ln AFL	0.079	0.079	0.074
ln SL–ln P_1_FL	0.056	0.063	0.045
ln SL–ln P_2_FL	0.058	0.053	0.054
ln SL–ln HL	0.034	0.031	0.022
ln SL–ln SNL	0.044	0.067	0.027
ln SL–ln OD	0.074	0.087	0.053
ln SL–ln IOW	0.066	0.059	0.065
ln SL–ln SOW	0.160	0.155	0.140
ln SL–ln POPW	0.050	0.058	0.060
ln SL–ln POL	0.057	0.044	0.035
ln SL–ln UJL	0.035	0.046	0.029
ln SL–ln LJL	0.034	0.046	0.033
ln SNL–ln POPW	0.052	0.097	0.068
SL–DFS counts	0.515	0.424	0.299
SL–DFR counts	0.649	0.607	0.420
SL–AFS counts	0.000	0.109	0.086
SL–AFR counts	0.626	0.629	0.581
SL–P_1_FR counts	0.624	0.589	0.432
SL–LLS counts	3.828	3.725	1.623
SL–SAL counts	1.117	0.614	0.837
SL–SBL counts	1.394	1.516	1.009
SL–UGR counts	1.020	1.131	0.659
SL–LGR counts	1.073	0.804	0.644
SL–TGR counts	1.366	1.507	0.963
SL–AV counts	0.155	0.279	0.191
SL–CV counts	0.420	0.370	0.336
SL–TV counts	0.426	0.414	0.333

## Discussion

### Growth-related morphological changes

The present study revealed that most body proportions of the three *Lateolabrax* species change with growth (Table 3). Although such proportional changes with growth have been reported for a number of fishes, including two black-and-white snappers of the genus *Macolor* (Kishimoto et al. 1987), Spanish mackerel, *Scomberomorusniphonius* (Yokogawa 1996), giraffe catfish, *Auchenoglanisoccidentalis* (Chioma et al. 2007), red porgy, *Pagruspagrus* (Minos et al. 2008), bluegill, *Lepomismacrochirus* (Yokogawa 2013a; Bell and Jacquemin 2017), largemouth bass, *Micropterussalmoides* (Yokogawa 2014), two flatfishes of the genus *Pleuronichthys* (Yokogawa 2015), and some sea banjofishes of the genus *Banjos* (Matsunuma and Motomura 2017), such have been frequently neglected, particularly in taxonomic studies.

**Figure 14.**
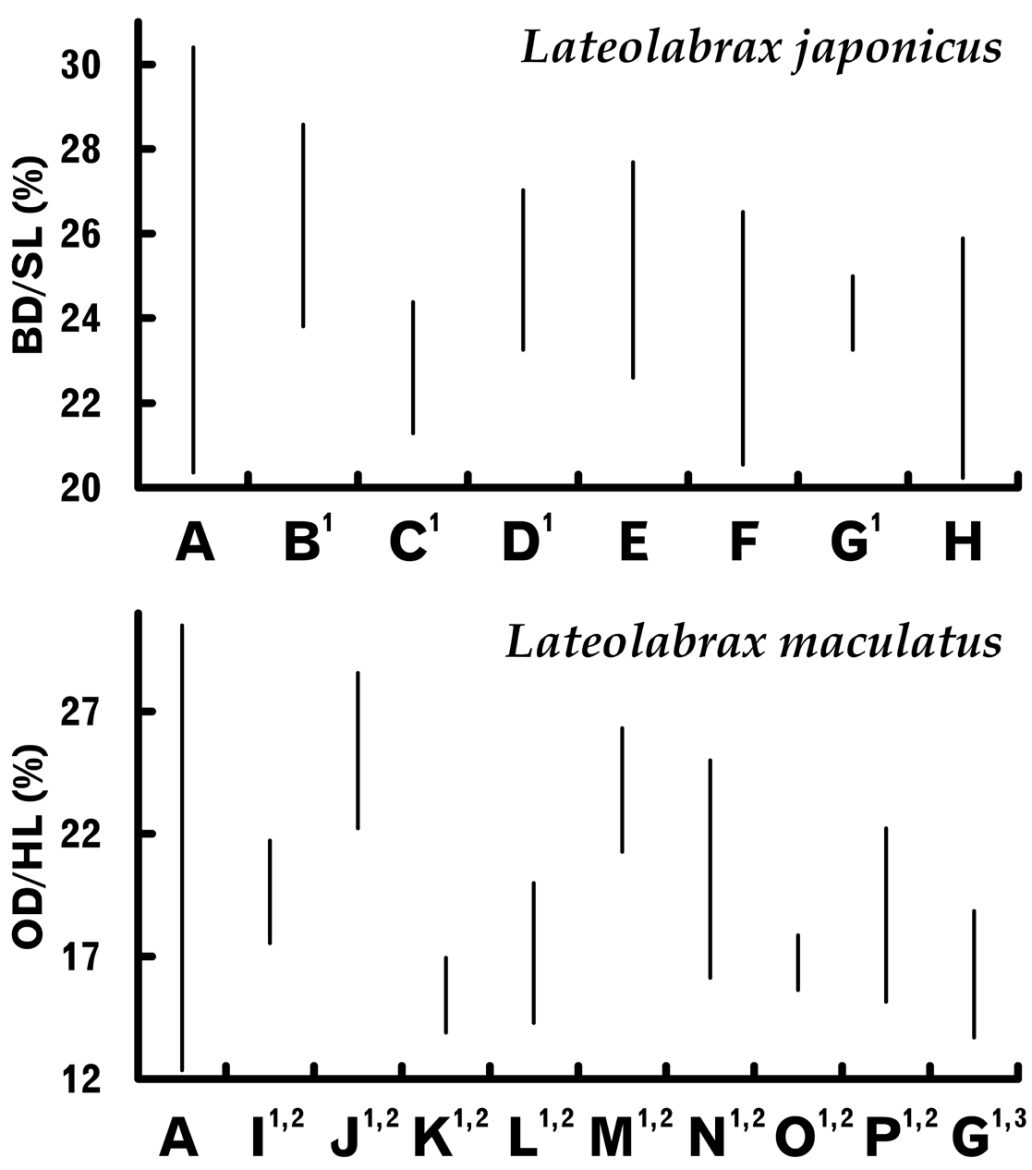
Proportional range comparisons of head length [HL, % of standard length (SL)] in *Lateolabraxjaponicus* (upper graph, axis labelled BD / SL) and orbital diameter (OD, % of HL) in *L.maculatus* (lower graph, axis labelled OD / HL) in the present study and previous literature. Data based on **A** present study **B**Katayama (1960a)**C**Lindberg and Krasyukova (1969)**D**Chyung (1977)**E**Yokogawa (1995)**F**Nozaka (1995)**G**Yamada et al. (2007)**H**Bae et al. (2016)**I**Chu et al. (1962)**J**Chu (1985)**K**Chen (1987)**L**Zheng (1987)**M**Chen et al. (1990)**N**Mao et al. (1991)**O**Gao (1991)**P**Cheng and Zhou (1997). ^1^ Proportional percentages were calculated as reciprocal numbers from proportional data (multiple numbers) therein given. ^2^ Despite descriptions as “*L. japonicus*,” synonymized as *L.maculatus* by Yokogawa (2013b). ^3^ Provisionally referred to as *Lateolabrax* sp., which was identical with *L.maculatus*.

On the other hand, taxonomic and related literature on *Lateolabrax* have commonly noted the diagnostic importance of ranges and / or averages of body proportions (e.g., Katayama 1960a, b; Yokogawa and Seki 1995; Kim and Jun 1997; Yamada et al. 2007; Murase et al. 2012), although such, being commonly subject to allometric growth, are largely biased by the body sizes of specimens examined. For example, Figure 14 summarizes proportional body depth (BD) and orbital diameter (OD) ranges previously reported for *L.japonicus* and *L.maculatus*, respectively, compared with the present study. The smaller proportional ranges previously reported were all less than those presented here, representing many variously-sized specimens, suggesting that the former were based on relatively few specimens. Also, the variations in published proportional ranges, in some cases showing no range overlap (e.g., Fig. 14J vs K; L vs M), suggested differing body size ranges of the material studied. Although such proportional data has often been included in taxonomic diagnoses, the inherent inconsistencies have made specimen comparisons and specific identifications problematic. In fact, the use of proportions subject to isometric growth in species diagnoses is a legitimate procedure, although such proportions are rare in both *Lateolabrax* species (Table 3) and the other species listed above. However, the use of non-isometric proportional data, traditionally under the premise of (presumed) isometric growth, in species diagnoses is inappropriate.

Differing growth-related proportional change patterns in the three *Lateolabrax* species include pre-anus length (PAL) (Fig. 4A–C, Table 3) and post-orbital preopercular width (POPW) (Fig. 6K, Table 3). Similarly, the very similar East Asian frog flounders *Pleuronichthyslighti* and *P.cornutus* have the caudal fin, and dorsal and anal fins shortened with growth in the former and latter, respectively (Yokogawa 2015), indicating the potential for differing specific patterns, even between closely related species. Comparisons of black bass congeners (genus *Micropterus*) have shown the upper jaw length proportion to increase with growth in *M.salmoides* (Yokogawa 2014), while remaining stable in *M.dolomieu* (Senou 2002). Although the three *Lateolabrax* species share a similar “bass shape” with *M.salmoides*, the upper and lower jaw length (UJL and LJL) / standard length (SL) proportions decreased with growth in the former (Fig. 6M, O, Table 3), unlike the latter (Yokogawa 2014). Also, it is notable that BD and head length (HL) proportions of the three *Lateolabrax* species decreased with growth (Fig. 5A, Table 3), in contrast to the centrarchids *M.salmoides* and *L.macrochirus* (Yokogawa 2013a), in which BD and HL increased with growth (Yokogawa 2014). This suggests that some phylogenetic factors may be responsible for growth-related proportional change patterns.

As in many other fishes (Okiyama 1988), BD of *L.japonicus* increased relatively with growth during the larval stage (from 13–16 to 26–30% of SL) until ca. 25 mm SL, thereafter being “stable,” according to Tanaka and Matsumiya (1982) and Tamura et al. (2013), although subsequently decreasing from ca. 30 to ca. 21% of SL (Fig. 5A). Similarly, HL of *L.japonicus* and *L.latus* increased relatively with growth during the larval stage (Kinoshita 1988), in contrast to the growth-related acute decrement of HL during the juvenile and adult stages (Fig. 4S, U). During the larval stage of *L.japonicus* and *L.latus* (11–19 mm SL), the greater HL / SL proportion of the latter compared with the former in same-sized larvae, enabled ready distinction of the two species from each other (Kinoshita 1988). Although a similar distinction was observed in juvenile fishes (ca. 40–100 mm SL), very similar growth-related HL decreasing patterns between the two species in the adult stage (Fig. 4S, U) made it clear that Kinoshita’s (1988) criterion for separation was applicable only for larvae of the two species.

Growth-related proportional change patterns of length-measured cephalic characters (based on SL and HL) were sometimes inconsistent in *L.japonicus* and *L.latus* (Fig. 6, Table 3), possibly due to HL being negatively allometric with SL (decreasing with growth) (Fig. 4S, U, Table 3) and paralleling or exceeding the change rate of some cephalic characters, resulting in negative allometry and isometry in SL-based relationships appearing as isometry and positive allometry in the HL-based ones, respectively. However, OD was negatively allometric relative to both SL and HL (Fig. 6C, D, Table 3), due to their degree of allometry relative to SL exceeding that of HL to SL. On the other hand, the consistency of the growth patterns between the two-way relationships in *L.maculatus* (Fig. 6A–P, Table 3) may be due to the growth-related decreasing rate of proportional HL being less apparent in this species (Fig. 4T) than in the others (Fig. 4S, U) and therefore less influential on the relative growth of the cephalic characters. Although HL-based proportions of cephalic characters have been frequently used for cephalic characters in taxonomic studies on *Lateolabrax* (e.g., literature cited in Fig. 14), it should be recognized that the base dimension (HL) is not a stable character.

The proportional values (percentages) of proportions subject to allometric growth are correlated with the base dimension (e.g., SL and HL). In Figure 14, because both BD and OD were negatively allometric in both *L.japonicus* and *L.maculatus* (Figs 5A, 6D, Table 3), high and low proportional values are regarded as representing small and large size specimens, respectively. McClelland (1844) noted in the original description of *L.maculatus* (as *Holocentrummaculatum*) that the eyes were large, indicating that his description was based on a small specimen(s). The OD / SL proportion taken from his specimen illustration (pl. 21, fig. 1) was 6.4%, whereas the SL calculated by the inverse function of the SL–OD / SL regression (Fig. 6C, Table 2) was ca. 184 mm, agreeing with the above suggestion. This suggests that length-measured characters (including OD) subject to allometric growth can be utilized for estimation of body size.

Hirota et al. (1999) compared their morphometric data for *L.maculatus* (as *Lateolabrax* sp.) from Kanto region, Japan [*n* = 6, 151–451 (average 298.3) mm SL] with those examined by Yokogawa and Seki (1995) [*n* = 62, 76.3–121.8 (average 97.6) mm SL], recording lower OD proportions (% of HL) for their specimens [18.5–25.3 (average 20.8) vs 21.3–30.5 (average 24.8)] (Hirota et al. 1999, table 1). Such inconsistency was clearly due to body size differences of the specimens examined in the two studies, i.e., the larger specimens in the former study provided lower OD proportions (Fig. 6D). Nevertheless, Hirota et al. (1999) suggested that the different OD proportions resulted from Yokogawa and Seki (1995) having measured eye diameter rather than OD, which was incorrect. Kim and Jun (1997) examined the morphology of Korean *L.japonicus* specimens from Kohung [*n* = 69, 77.4–353.0 (average 175.0) mm SL] and Puan [*n* = 6, 465.0–640.0 (average 582.0) mm SL], giving similar average proportional values (% of SL) for BD (25.8 and 24.3), caudal peduncle depth (CPD) (31.6 and 32.1), HL (31.4 and 31.8) and OD (19.7 and 19.8) for the respective lots (Kim and Jun 1997, table 1). However, those degrees of proportional similarity between such different-sized specimens is extremely unlikely due to the highly negatively allometric proportions of those characters in this species (Figs 5A, B, 4S, 6C, Table 3).

Because most of the length-measured characters of the three *Lateolabrax* species were subject to allometric growth (Table 3), raw dimension measurement data were logarithm-transformed in order to transform the data distribution to be symmetric for statistical analysis, including canonical discriminant analysis (Bae et al. 2016) and analysis of covariance (ANCOVA), performed in the present study. Although Wang et al. (2016) provided multiple-regression analyses between body weight (BW) and some body dimensions for *L.maculatus* using raw data, the approach was problematic, because the raw dimension data (including BW) needed to have been logarithm-transformed before analysis, as done for *M.salmoides* by Yokogawa (2014).

Counts of pored scales on the lateral line (LLSs) and scales above the lateral line (SALs) tended to increase and decrease with growth, respectively, in *L.japonicus* (Fig. 9A, D, Table 4), those of scales below the lateral line (SBLs) and lower-limb and total gill rakers (LGRs and TGRs) tending to increase with growth in *L.maculatus* (Fig. 9H, K, N, Table 4). By contrast, overall meristic counts (except dots) did not change with growth in *L.latus* (Table 4), implying some phylogenetic determination of growth-related meristic characters, as in the case of PSAL change patterns. Although the mechanism by which such counts increase or decrease with growth is uncertain, an SBL count increase with growth has been reported for *L.macrochirus* (Yokogawa 2013a), *M.salmoides* (Yokogawa 2014) and *P.cornutus*, in which gill raker numbers also increased with growth (Yokogawa 2015), suggesting that such phenomena are not so rare in fishes. Although meristic characters have been frequently used as important keys in taxonomic studies on the premise that they are stable at any body size, the potential for growth-related changes should be considered and actively assessed in taxonomic studies.

Nozaka (1995) examined the morphology of *L.japonicus* fingerlings from the eastern Seto Inland Sea (*n* = 112, average 141.1 mm SL), comparing his data with Yokogawa and Seki (1995) [*n* = 65, 122.8–417.0 (average 301.4) mm SL] and noting differences in LLS and gill raker numbers (average LLSs = 76.4 and 83.1, average TGRs = 24.9 and 27.2, in the former and latter, respectively). Inconsistency in LLS counts may have resulted from body size differences in specimens examined, larger specimens resulting in higher LLS counts (Fig. 9A, Table 4). On the other hand, the difference in gill raker counts, which do not change with growth in *L.japonicus* (Fig. 9J, M, Table 4), may have resulted from the non-inclusion of rudiments located on the gill arch edges, since low gill raker counts as reported by Nozaka (1995) have not been found in the many other *L.japonicus* samples examined from around Japan (Yokogawa, unpublished data).

The growth-related status of dots / spots on the lateral body region also varied among the three *Lateolabrax* species. In *L.japonicus* and *L.latus*, although dots appeared in some smaller specimens (up to 260.6 and 254.8 mm SL, respectively), they disappeared with growth (Fig. 11A, C), a well-known phenomenon in the former species (e.g., Katayama 1960a, 1960b; Yokogawa 1995; Kim and Jun 1997; Kim et al. 2004; Ishikawa and Senou 2010), but barely noted in taxonomic descriptions of the latter species, other than Katayama (1957, 1960a) and Murase et al. (2012). This may have been due to such dots being so fine or faint (Fig. 2C) that they were overlooked, or because descriptions were based only on large specimens. However, spot counts were not related to body size in *L.maculatus*, which typically had many clear spots in both large and small specimens (Fig. 2B, Table 4). Although many taxonomic descriptions of this species have incorrectly noted that spot counts decreased gradually with growth (Tchang et al. 1955; Chu et al. 1962, 1963; Chu 1985; Chen 1987; Chen et al. 1990; Li and Zhang 1991; Feng and Jiang 1998; Chen and Fang 1999; Wang et al. 2001; Zhao and Zhong 2006), such may have been based only on subjective observations without statistical analysis, unlike the present study. On the other hand, large individuals of this species tend to have smaller and more rounded (non-jagged) spots than in small individuals [e.g., Katayama 1984, plate 108-I, 52 cm, as a variation of *L.japonicus*; Yokogawa et al. 1996, fig. 1, 600 mm in total length (TL), as *L.* sp.], which may have provided some grounds for the above views. Descriptions of *L.maculatus* (as *L.japonicus*) from Hong Kong noted that in young specimens, spots were larger and fewer in number, whereas with advancing fish age the large spots become smaller and more numerous (Chan and Tang 1968; Sadovy and Cornish 2000). However, although growth-related spot size decrement is correct, growth-related spot number increment is not.

The proportional growth-related change pattern of pectoral scaly area length (PSAL) in *L.latus* closely fitted a power regression (Fig. 4X, Table 2). However, simple patterned regressions could not be applied to *L.japonicus* and *L.maculatus* since they exhibited inverted V-shaped changes (Fig. 4V, W). This may reflect the phylogenetic status of the three species, *L.latus* being genetically further from the other two species (Yokogawa 1998; Song et al. 2008; Shan et al. 2016). A similar growth-related change pattern was also observed for the maximum blotch diameter on the dorsal fin (% of SL) in *Banjosbanjosbanjos* (Matsunuma and Motomura 2017, fig. 8d), inferring that such non-linear patterns arise in some characters in which dimensions are not determined by internal bony structure, rather than in normal body portions. Although PSAL, as defined by Yokogawa and Seki (1995) (see above), was examined in *L.japonicus* and *L.maculatus*, overall growth-related change patterns were limitedly revealed for both at that time due to size-biased samples. Accordingly, Nozaka’s (1995) examination of *L.japonicus* fingerlings (see above) resulted in a much smaller proportional PSAL range and average than those given by Yokogawa and Seki (1995) for larger examples of that species. Such disagreement was regarded as arising from body size differences in the material specimens between the two studies.

### Inter-specific differences and taxonomy

*Lateolabraxlatus* is typically characterized by a deeper body, represented by BD and CPD. However, neither character provides unequivocal identification due to the range overlap for proportional BD and CPD between *L.latus* and *L.japonicus* (Katayama 1957, 1965). In the present study, although the scatter plots for proportional BD and CPD of *L.latus* were well separated from those of the other two species, some overlap occurred in the smaller size class (< ca. 200 mm SL) (Fig. 5A, B). However, the newly defined dimension caudal peduncle anterior depth (CPAD), located between BD and CPD (Fig. 3), is suitable for distinguishing *L.latus* from the other two species, there being no plot overlap with the latter (border level 15%) (Fig. 5C).

The CPAD proportion may be a useful feature for specific identification, since it can also be determined from illustrations and photographs of *Lateolabrax* species. For instance, an illustration of “*L. japonicus* (as *Perca-labrax japonicus*)” in Fauna Japonica (Temminck and Schlegel 1846, pl. II, fig. 1, drawn by Keiga Kawahara) may, in fact, be *L.latus*, because the proportional CPAD (% of SL) measured from the illustration was 15.4%, falling within the range of the latter (Fig. 5C). Because the SL of the illustrated specimen estimated by the earlier-described procedure (use of an inverse function of SL–OD / SL regression for *L.latus*) was ca. 336 mm, proportional BD and CPD (% of SL), which had no plot overlap with the larger size classes (>200 mm SL) of the other two *Lateolabrax* species, may also be used for specific identification. The proportional BD and CPD of the illustrated specimen were 29.3 and 12.1%, respectively, corresponding with the ranges of *L.latus* (Fig. 5A, B). Although Katayama (1960b) also recognized the greater BD and CPD proportions of Temminck and Schlegel’s (1846) specimen, he identified it as *L.japonicus* because the dorsal and anal fin ray counts (xiv, 13 and iii, 8, respectively) corresponded to the ranges for *L.japonicus*. In fact, he may have counted 12 spines in the first dorsal fin, and 2 spines plus 13 rays in the second (SDF). However, the SDF should be regarded as comprising 1 spine and 14 rays, the ray next to the first SDF spine having a distal branch. Specimens examined in the present study included 6 *L.latus* with 14 dorsal fin rays (DFRs) and 8 anal fin rays (AFRs), supporting the opinion that Temminck and Schlegel’s (1846) illustration was of *L.latus*. Similarly, illustrations of *L.japonicus* and *L.latus* in Katayama (1965, figs 520 and 521) should actually be reversed, since their proportional CPAD (% of SL) values were 15.1 and 13.5%, respectively, falling within the respective ranges of *L.latus* and *L.japonicus*.

In addition to caudal peduncle stoutness in *L.latus*, Hatooka (2000, 2013) proposed peduncle shortness as a diagnostic character of the species. Similarly, Murase et al. (2012) recorded proportions of caudal peduncle length (CPL) (% of SL) for *L.latus* (*n* = 27, 18.7–20.9), *L.japonicus* (*n* = 25, 20.0–23.4), and *L.maculatus* (*n* = 7, 20.7–22.3), indicating a clear difference between *L.latus* and the other two species. However, despite the distinctly downward shift in plot distribution in *L.latus* from the other two species found here, the CPL proportion range (*n* = 136, 18.3–22.7) largely overlapped those of *L.japonicus* (*n* = 229, 18.5–24.6) and *L.maculatus* (*n* = 170, 18.6–25.3), owing to considerable variation in plot distribution in the latter two species (Fig. 4G–I). The disagreement between the above two studies and the present one is likely to have resulted from differing numbers of specimens examined. In conclusion, although the proportional CPL of *L.latus* tended to be lower than in the other species, adoption of the feature as a diagnostic key for *L.latus* is problematic.

Caudal fin notch depth (CFND) has been recently proposed as a new character for distinguishing *L.latus* from the other two species, the former having a shallower CFND than the others (Hatooka 2000, 2013). However, although growth-related patterns of proportional CFND (% of SL) differed from one another among the three species (Fig. 4J–L) and ANCOVA for the logarithm-transformed regressions indicated significant differences of the slopes between any two species (Table 6), the ranges relative to overall SL (2.9–7.9, 2.0–8.4 and 1.9–7.4% for *L.latus*, *L.japonicus* and *L.maculatus*, respectively) were similar (Fig. 4J–L) and unable to distinguish between species. In fact, the proportional CFND of *L.latus* decreased acutely with growth, with relatively little variation owing to high correlation with SL (Fig. 4L, Table 3), being almost stable at low values (around 4–5%) in specimens > ca. 200 mm SL (Fig. 4L). In contrast, the other two species had highly variable proportional CFND, up to ca. 8% at any body size (Fig. 4J, K). Therefore, individual specimens of *L.japonicus* and *L.maculatus* with greater CFND may give the impression that *L.latus* has a shallower CFND than the others, as emphasized by some photographs of *L.latus* in which the caudal fins are so well opened that CFND decreases considerably (nearly truncate) (e.g., Masuda et al. 1975, pl. 42E; Ishikawa and Senou 2010; Murase et al. 2012, fig. 2C). It is possible that the caudal fin of *L.latus* may spread more than that of the other two species owing to broader membrane between the fin rays (Fig. 1E, F), particularly when fresh (when specimens were photographed). Notwithstanding, the results herein clearly indicate that CFND is problematic as a key character. Although Shimose et al. (2011) made underwater observations of and photographed a single *Lateolabrax* fish at Ishigaki Island, Okinawa, Japan, suggesting it to likely be *L.latus* based on some visually-recognized features, including CFND, the influence of such a key in the popular media is unfortunate.

Among the length-measured cephalic characters of *L.latus*, plot separation of that species from the others was marked for snout length (SNL) (Fig. 6A, B), post-orbital length (POL) (Fig. 6K, L), and upper and lower jaw lengths (UJL and LJL) (Fig. 6M–P). In particular, SNL may be a practical means of distinguishing *L.latus* from the others because plots were vertically separated for both in the SL- and HL-based relationships (border levels ca. 9 and 28%, respectively) (Fig. 6A, B), which were similar to Murase et al.’s (2012) results. However, POL may not be practical for identification because the plots and vertical axis ranges overlapped considerably with those of *L.japonicus* (Fig. 6K, L). Although Murase et al. (2012) showed an unequivocal difference in POL (% of SL) between *L.latus* (*n* = 27, 14.1–15.8) and the other species [*L.japonicus* (*n* = 25, 16.1–18.5), *L.maculatus* (*n* = 7, 16.4–20.2)], such may have been due to the low numbers specimens examined, as in the case of CPL. The fact that SNL and POL of *L.latus* are greater and shorter, respectively, than in the other species infers that the eyes of *L.latus* are located more posteriorly than in the latter.

The UJL and LJL plots for all three species (SL-based relationships) were well clustered around their regression curves (high negative allometry), but could not be distinguished from one another vertically (Fig. 6M, O). On the other hand, since the UJL and LJL plots of *L.latus* in the HL-based relationships formed almost horizontal clusters, they could be vertically distinguished from those of the other two species (border levels of ca. 45 and 49%, respectively) (Fig. 6N, P). Despite Murase et al.’s (2012) proposal of some diagnostic characters for *L.latus* including greater SNL and shorter POL, they excluded UJL, despite having measured that dimension. Although Hirota et al.’s (1999) (see above) examination of *L.maculatus* recorded SNL and UJL proportions (% of HL) [23.2–30.0 (average 26.3) and 39.4–46.4 (average 42.5), respectively], the maximum values of both fell within the ranges peculiar to *L.latus* (Fig. 6B, N). Assuming correct calculations, their catalogued “*L. maculatus*” specimens (whereabouts unknown) may have included *L.latus*. This possibility is also suggested by their higher counts of DFRs [13–14 (average 13.3)] and AFRs [8–9 (average 8.2)], including a small proportion of specimens (*n* = 6) with minor counts in *L.maculatus* [14 DFRs (16.6%) and 9 AFRs (5.3%)] (Fig. 10B, D).

The original description of *L.latus* included several diagnostic meristic characters, including counts of DFRs, AFRs and SBLs (Katayama 1957). In particular, DFR numbers =15, considered peculiar to the species, have subsequently been noted as an important diagnostic key (Katayama 1960a, 1965, 1984; Masuda et al. 1975; Araga 1981; Hatooka 1993). However, because some *L.latus* specimens with 14 DFRs (overlapping the ranges of the other two *Lateolabrax* species) have been recognized (Sakai et al. 1998; Hatooka 2000, 2013; Murase et al. 2012), including 7.4% of *L.latus* specimens in the present study, DFR counts alone cannot absolutely distinguish *L.latus* from the others, although higher DFR counts may be useful (Fig. 10B). In contrast, AFR and SBL counts have rarely been adopted as diagnostic for *L.latus*, inferring that the count range overlaps between *L.latus* and the other two species are problematic for specific identification. In the present study, *L.latus* was well separated from the other species by AFRs (Fig. 10D) and DFRs, whereas SBL counts broadly overlapped (Fig. 10H). On the other hand, pectoral fin ray (P_1_FR) counts, which have not been emphasized as having taxonomic significance for *L.latus*, showed a strong modal shift between *L.latus* and *L.japonicus* (16 and 17, respectively) (Fig. 10E). Although the large range overlap of P_1_FR counts in *L.japonicus* and *L.maculatus* preclude their diagnostic use, they may be useful in the case of *L.latus*. For example, the two *Lateolabrax* specimens collected from Tanegashima Island both having 16 P_1_FRs (Sakai et al. 1998) are likely referable to *L.latus*.

In addition to length-measured and meristic characters in the original description of *L.latus* a further diagnostic feature proposed was the possession of ventromandibular scale rows (VSRs) (Katayama 1957). Although frequently noted as diagnostic for *L.latus* until recent years (e.g., Katayama 1960a, 1965, 1984; Masuda et al. 1975; Araga 1981; Hatooka 1993), the possession of such scales has subsequently been omitted from keys to the genus *Lateolabrax* (Hatooka 2000, 2013) owing to the presence of VSRs in some specimens of *L.japonicus* and *L.maculatus* (Table 5) (Paxton and Hoese 1985; Hirota et al. 1999; Kang 2000; Murase et al. 2012). Furthermore, the lack of VSRs in some small *L.latus* (mainly ≤100 mm SL) (Table 5) underlines the unsuitability of this feature as a diagnostic character for *L.latus*. It was clear in the present study that VSRs did not exist in larvae and juveniles of all *Lateolabrax* species, but first appeared in *L.latus* at ca. 90 mm SL, thereafter rapidly developing with growth until present in almost all large individuals. In *L.japonicus* and *L.maculatus*, the appearance of VSRs was delayed, beginning from around 150 and 200 mm SL, respectively, and thereafter gradually developing with growth, although still absent in some large individuals. Such specific differences in squamation development may be common for PSAL (Fig. 4X) and dorsocephalic scale rows (DSRs) (Fig. 12), development being greatest in *L.latus* and least in *L.maculatus*, as indicated by Murase et al. (2012).

The diagnosis accompanying the original description of *L.latus* included ventral (pelvic fins) generally dusky, unlike in *L.japonicus* (Katayama 1957), followed by Katayama (1965) and Araga (1981). Although such coloring was infrequent in preserved *L.latus* specimens examined here, it has been noted in some large fresh adult specimens [e.g., photographs in Araga (1981) and Ishikawa and Senou (2010)]. However, non-dusky (pale) pelvic fins have been commonly observed in small *L.latus* (to fingerling size) (Fig. 1E, Murase et al. 2012, fig. 2A, B) and some large fresh condition specimens (Fig. 1F, Murase et al. 2012, fig. 2C). Possibly based on this supposed feature, the English name “blackfin sea bass” has been employed for *L.latus* (e.g., Matsuyama et al. 2002; Arakaki et al. 2014; FishBase 2018), however, such naming is not suitable, because it suggests that all fins were black, and many *L.latus* specimens including the large individual (915 mm TL) figured in FishBase (2018) do not have dusky (“black”) pelvic fins. Instead, “flat sea bass,” which describes the deeper body, a common feature of the species, should be applied for *L.latus*, following Yokogawa and Kishimoto (2012).

Recent keys for identification of *L.japonicus* and *L.maculatus* have adopted SNL, that of *L.maculatus* supposedly being relatively shorter than that of the former (Yamada et al. 2007; Hatooka 2000, 2013). However, plots of proportional SNL largely overlapped in smaller size classes (< ca. 200 mm SL) of the two species, although plots for *L.maculatus* shifted downward (highly negative allometry) and were clearly separated from those of *L.japonicus* in specimens > ca. 200 mm SL (border levels ca. 7.7% and 24% for SL- and HL-based relationships, respectively) (Fig. 6A, B). Accordingly, SNL proportions enable separation only of large specimens (> ca. 200 mm SL) of the two species; e.g., Wakabayashi and Nakamura’s (2003)*L.maculatus* specimen (as *L.* sp.) from Shima Peninsula, Japan (381 mm SL) was identifiable by its SNL proportions (7.1 and 22.8% of SL and HL, respectively).

On the other hand, post-orbital preopercular width (POPW) is a notable dimension, showing a contrasting pattern to SNL, i.e., plots of proportional POPW in small (< 200 mm SL) *L.maculatus* shifted upward and separated completely from those of similar sized *L.japonicus* (border levels ca. 7.5% and 23% for SL- and HL-based relationships, respectively), although larger specimens (> 200 mm SL) of the two species had some overlap due to the relative decrease of POPW with growth (highly negative allometry) in the former (Fig. 6I, J). Thus, a combination of SNL and POPW proportions [for small (< ca. 200 mm SL) and large (> ca. 200 mm SL) specimens, respectively] enables the two species to be separated unequivocally for their entire size range. Furthermore, the POPW / SNL proportion, which largely separates the two species throughout their entire size range (border level 90%) (Fig. 8), can also be adopted.

Proportional differences between *L.japonicus* and *L.maculatus* were also apparent in many of the fin lengths (first and second dorsal, caudal and pectoral), proportions of the former being distinctly greater than those of the latter in smaller specimens (< ca. 200 mm SL), although plots of the two species overlapped in the larger size class (> ca. 200 mm SL), due to the relative fin lengths decreasing and not changing with growth in the former and latter species, respectively (Fig. 5E–H). That this means of distinguishing between small specimens of *L.japonicus* and *L.maculatus* has largely gone unrecognized is probably due to a lack of morphological examination of small *Lateolabrax* specimens. The benchmark size of 200 mm SL being common to SNL, POPW and fin lengths of the two species suggests some synchronization of specific growth-related morphological changes.

Although Yokogawa and Seki (1995, figs 6, 7) proposed that considerable differences in LLS and gill raker numbers were sufficient for unequivocal differentiation of *L.japonicus* and *L.maculatus* when used in combination, the present study has demonstrated greater count range overlaps between the two species (70–84 LLSs and 24–26 TGRs, vs 76–82 LLSs and 24 TGRs) (Fig. 10F, K), due to LLS and gill raker counts increasing with growth in *L.japonicus* and *L.maculatus*, respectively (Fig. 9A, M, Table 4). Similarly, Kang’s (2000) comparable frequency distributions of LLS and gill raker counts between the two species from Korean waters may have resulted from a size bias in specimens examined, his *L.maculatus* material including only very large specimens (ca. 500–750 mm SL). Accordingly, counts of LLSs and gill rakers, which can be biased by specimen size, are now likely to be unsuitable for distinguishing between the two species. In fact, Lou et al. (2002), who compared morphology between *L.japonicus* (1 sample lot from Tokyo, Japan) and *L.maculatus* (5 sample lots from Beihai, Xiamen, Fuzhou, Zhoushan and Weihai, China), showed considerable range overlaps for LLS and TGR counts, although the average values of those counts for *L.maculatus* were unequivocally lower than those for *L.japonicus*. Although Iseki et al. (2010) identified 263 *Lateolabrax* specimens from western Japan as *L.maculatus* (as *L.* sp.) based on LLS and gill raker counts proposed by Yokogawa and Seki (1995), some difficulties in identification may have been encountered due to some of their specimens being very large (up to 1130 mm SL), with gill raker counts that approached or overlapped the range for *L.japonicus*.

On the other hand, caudal and total vertebral counts (CV and TV, respectively), in which dominant counts were almost completely replaced between *L.japonicus* and *L.maculatus* (20 and 19 CVe, 36 and 35 TVe, for the former and latter, respectively) (Fig. 10M, N), may be useful for specific identification because they do not change with growth (Table 4). A modal count of 35 TVe in *L.maculatus* was indicated by Lou et al. (2002) (see above), who recorded average TV counts for 5 sample lots from China, viz., 34.75 (Beihai, *n* = 40), 34.64 (Xiamen, *n* = 19), 34.90 (Fuzhou, *n* = 10), 34.98 (Zhoushan, *n* = 27) and 35.07 (Weihai, *n* = 50), in spite of a geographic cline that suggested a trend towards lower and higher TVe in sample lots from southern and northern regions, respectively. Notwithstanding, Chen et al. (2001) recorded an average TV count of 35.31 (*n* = 98) for a sample lot from Laizhou, China, inferring that approximately 30% of their specimens had 36 TVe, which largely contradicts the present results (Fig. 10N). However, the former average count is suspect, differing considerably from the sample lot from Weihai (Lou et al. 2002), located close to Laizhou. In fact, such a high average TV value has not been recorded elsewhere at any time for *L.maculatus* (Yokogawa and Seki 1995; Yokogawa et al. 1996; Lou et al. 2002). Although vertebral counts [abdominal (AV), CV and TV, respectively] of *L.japonicus* and *L.latus* are similar to each other, those of *L.maculatus* stand apart (Fig. 10L–N, Table 7), in contrast to their phylogenetic relationship (Yokogawa 1998; Song et al. 2008; Shan et al. 2016). In this case, since the difference in *L.maculatus* was primarily due to a difference in CV counts, which generally reflect inter-specific differences or lower, unlike AV counts which may reflect differences at a higher taxonomic level (Takahashi 1962), the vertebral count peculiarity in *L.maculatus* may not have phylogenetic significance.

Although *L.maculatus* typically possessed many black spots on the body, individual spot counts and patterns varied considerably (Yokogawa 2013b, fig. 2), a few specimens (4.9% of total) entirely lacking spots. In addition, the proportion of dotted *L.japonicus* specimens (35.6% of total) made visual separation of the two species difficult, the use of color pattern for specific identification being of value only as an accessory character. Youn’s (2002) key, however, distinguished between the two species on the presence or absence of black spots, may causing mis-identification.

Yokogawa and Seki (1995) demonstrated differences between *L.maculatus* and *L.japonicus* in some newly-demonstrated characters, including PSAL and DSRs (scale development in these characters being poorer in *L.maculatus*). However, because their examined material was size-biased (see above), overall growth-related change patterns were still unclear. Examination of PSAL and DSR in the present study have overcome that problem. Although differences between the two species were apparent in specimens < ca. 150 mm SL, squamation developed thereafter with growth in *L.maculatus*, the two species consequently having similar degrees of squamation in large specimens (Figs 7, 12). Notwithstanding, specific differences in specimens < ca. 150 mm SL can be used to identify *Lateolabrax* individuals up to fingerling size. Growth-related squamation development has been examined in laboratory-reared larval and juvenile *L.japonicus* (Fukuhara and Fushimi 1982) and *L.maculatus* (Kang 2000). Although squamation initially occurred on the caudal peduncle at ca. 19 mm SL in both species, body squamation was completed earlier in the former (ca. 35 mm SL vs 47 mm SL) (Fukuhara and Fushimi 1982; Kang 2000), indicating delayed development in *L.maculatus*. The slower development in PSAL and DSRs in *L.maculatus* might be an extension of such squamation delay, which is a characteristic peculiar to that species.

A morphological difference in the first anal pterygiophore (FAP) between *L.japonicus* and *L.maculatus* was initially noted by Kang (2000) during his detailed osteological observations of the three *Lateolabrax* species, and included in one of his keys (for adults) to the genus *Lateolabrax*; FAPs were arched and straight in *L.japonicus* and *L.maculatus*, respectively (Kang 2000). However, FAPs of small *L.japonicus* specimens (< ca. 90 mm SL) were found here to be straight (Fig. 13A), a condition not found by Kang (2000) due to his examining only larger specimens (minimum size 185.5 mm TL). Although Kang (2000) also described FAP in *L.latus* as straight, some examples of that species examined here had the FAP slightly arched distally (Fig. 13J, L). Because Kang (2000) examined only three *L.latus* specimens, ontogenetic morphological variations were not considered at that time. However, despite the growth-related morphological changes now apparent in *L.japonicus*, morphological differentiation of FAP is stable in specimens of *L.japonicus* and *L.maculatus* > 90 mm SL (Fig. 13B–D, F–H), enabling separation of the two species. Yokogawa and Kishimoto’s (2012) identification of a long-finned *Lateolabrax* specimen from Japan (SPMN-h 40001, 331 mm SL) as *L.japonicus* was based on its genetic characteristics, although morphological identification of the specimen was equivocal, the TV count of 35 being suggestive of *L.maculatus* (Fig. 10N). However, identification of the specimen as *L.japonicus* was settled by the FAP being arched (Yokogawa and Kishimoto 2012, fig. 2a).

Standard errors (SEs) for the length-measured and meristic character regressions, which indicated degrees of morphological variation, were generally lowest in *L.latus* (Table 8), suggesting less morphological variation in that species. This may be due to less genetic variation, average observed heterozygosity for 28 isozymic loci in *L.latus* being 0.033, much lower than that of *L.japonicus* (0.095) and *L.maculatus* (0.103) (Yokogawa 1998). Usually, lower genetic diversity occurs in a small or reduced population, but the *L.latus* specimens examined in the present study were from a broad area around southern Japan. Possibly, in spite of the species’ broad distribution, *L.latus* resources may not be so abundant, since the species is much less popular than *L.japonicus* in Japanese commercial markets. In contrast, SEs were generally highest in *L.maculatus* (Table 8), inferring considerable morphological variation. The significant geographical differences in otolith morphology among some *L.maculatus* samples from China (Ye et al. 2007) may have also resulted from its genetic diversity. This is supported by *L.maculatus* being broadly distributed along the east Asian coast, with some local populations being so genetically divergent from one another as to form a genetic / geographic cline, unlike *L.japonicus*, which is genetically stable (Yokogawa 2004; Liu et al. 2006; Han et al. 2015). In this regard, Zhao et al. (2018) reasonably considered that the Leizhou Peninsula, Hainan Island and Shandong Peninsula were major physical barriers, substantially blocking gene flow and genetic admixture among local *L.maculatus* populations.

The present study demonstrated a number of growth-related morphological changes in the three *Lateolabrax* species, including some new key characters for identification. Despite the number of taxonomic descriptions and studies of *Lateolabrax*, such features have remained obscure due to the limited numbers of specimens examined and an inherent belief that fish morphology is stable regardless of growth, notwithstanding some recent unique allometric approaches to fish morphology and taxonomy (e.g., Sidlauskas et al. 2011). The importance of investigating possible growth-related morphological changes, as well as meristic characters, is emphasized herein, as an understanding of proportional changes throughout the overall size range of a species may provide certain criteria which can distinguish between species and become keys for identification. Although such examinations need to be based on many specimens of various sizes, it may not be so difficult for commercial fishes, including *Lateolabrax*. Based on the results of the present study, a new key to the genus *Lateolabrax* is proposed.

### Key to *Lateolabrax* species

**Table:** 

a^1^	Caudal peduncle anterior depth [% of standard length (SL)] > 15%. Snout length (% of SL) > 9%. Upper and lower jaw length [% of head length (HL)] > 45% and 49%, respectively. Dorsal fin rays 15–16 [rarely 14 (7.4%)]. Anal fin rays 9 (usually)–11 [rarely 8 (11.0%)]	*** Lateolabrax latus ***
a^2^	Caudal peduncle anterior depth (% of SL) ≤ 15%. Snout length (% of SL) ≤ 9%. Upper and lower jaw length (% of HL) ≤ 45% and 49%, respectively. Dorsal fin rays 14 or fewer. Anal fin ray counts 8 or fewer (rarely 9)	**b**
b^1^	Post-orbital preopercular width (POPW) [% of snout length (SNL)] < 90% [POPW (% of SL) < 7.5% in specimens ≤ 200 mm SL; SNL (% of SL) > 7.7% in specimens > 200 mm SL]. Caudal vertebrae 20 (usually)–21 [rarely 19 (13.5%)]; total vertebrae 36 (usually)–37 [rarely 35 (13.5%)]. First anal pterygiophore modestly arched in specimens ≥ 90 mm SL. Spots / dots absent on body in specimens > 260 mm SL (although some specimens ≤ 260 mm SL have some dots restricted to upper part than lateral line)	*** Lateolabrax japonicus ***
b^2^	Post-orbital preopercular width (POPW) [% of snout length (SNL)] ≥ 90% [POPW (% of SL) ≥ 7.5% in specimens ≤ 200 mm SL; SNL (% of SL) ≤ 7.7% in specimens > 200 mm SL]. Caudal vertebrae 18–19 (usually) [rarely 20 (9.2%)]; total vertebrae 34–35 (usually) [rarely 36 (6.6%)]. First anal pterygiophore straight. Usually many clear black spots on lateral and dorsal body regions (usually even on lower part than lateral line)	*** Lateolabrax maculatus ***
